# The Two-Component System CopRS Maintains Subfemtomolar Levels of Free Copper in the Periplasm of Pseudomonas aeruginosa Using a Phosphatase-Based Mechanism

**DOI:** 10.1128/mSphere.01193-20

**Published:** 2020-12-23

**Authors:** Lorena Novoa-Aponte, Cheng Xu, Fernando C. Soncini, José M. Argüello

**Affiliations:** a Department of Chemistry and Biochemistry, Worcester Polytechnic Institute, Worcester, Massachusetts, USA; b Instituto de Biología Molecular y Celular de Rosario, Universidad Nacional de Rosario, Consejo Nacional de Investigaciones Científicas y Técnicas, Rosario, Santa Fe, Argentina; University of Iowa

**Keywords:** *Pseudomonas aeruginosa*, copper, homeostasis, periplasm, two-component regulatory systems

## Abstract

Two-component systems control periplasmic Cu^+^ homeostasis in Gram-negative bacteria. In characterized systems such as Escherichia coli CusRS, upon Cu^+^ binding to the periplasmic sensing region of CusS, a cytoplasmic phosphotransfer domain of the sensor phosphorylates the response regulator CusR. This drives the expression of efflux transporters, chaperones, and redox enzymes to ameliorate metal toxic effects. Here, we show that the Pseudomonas aeruginosa two-component sensor histidine kinase CopS exhibits a Cu-dependent phosphatase activity that maintains CopR in a nonphosphorylated state when the periplasmic Cu levels are below the activation threshold of CopS. Upon Cu^+^ binding to the sensor, the phosphatase activity is blocked and the phosphorylated CopR activates transcription of the CopRS regulon. Supporting the model, mutagenesis experiments revealed that the Δ*copS* strain exhibits maximal expression of the CopRS regulon, lower intracellular Cu^+^ levels, and increased Cu tolerance compared to wild-type cells. The invariant phosphoacceptor residue His_235_ of CopS was not required for the phosphatase activity itself but was necessary for its Cu dependency. To sense the metal, the periplasmic domain of CopS binds two Cu^+^ ions at its dimeric interface. Homology modeling of CopS based on CusS structure (four Ag^+^ binding sites) clearly supports the different binding stoichiometries in the two systems. Interestingly, CopS binds Cu^+/2+^ with 3 × 10^−14^ M affinity, pointing to the absence of free (hydrated) Cu^+/2+^ in the periplasm.

**IMPORTANCE** Copper is a micronutrient required as cofactor in redox enzymes. When free, copper is toxic, mismetallating proteins and generating damaging free radicals. Consequently, copper overload is a strategy that eukaryotic cells use to combat pathogens. Bacteria have developed copper-sensing transcription factors to control copper homeostasis. The cell envelope is the first compartment that has to cope with copper stress. Dedicated two-component systems control the periplasmic response to metal overload. This paper shows that the sensor kinase of the copper-sensing two-component system present in *Pseudomonadales* exhibits a signal-dependent phosphatase activity controlling the activation of its cognate response regulator, distinct from previously described periplasmic Cu sensors. Importantly, the data show that the system is activated by copper levels compatible with the absence of free copper in the cell periplasm. These observations emphasize the diversity of molecular mechanisms that have evolved in bacteria to manage the copper cellular distribution.

## Abstract (precis)

Copper is a micronutrient required as cofactor in redox enzymes. When free, copper is toxic, mismetallating proteins and generating damaging free radicals.

## INTRODUCTION

Copper is a cellular micronutrient required for redox enzymatic functions (1, 2). However, free Cu undergoes deleterious Fenton reactions, metallates noncognate binding sites, and promotes disassembly of Fe-S centers (3, 4). Early studies in the field took advantage of Cu toxicity to identify widely distributed proteins conferring metal tolerance, namely, metal-sensing transcriptional regulators and efflux transporters (1, 4–7). Recent studies have, however, started to uncover regulated distribution systems that move the metal among cellular compartments and target Cu^+^ to cognate metalloproteins while maintaining the required homeostasis (8–15). These include Cu^+^-sensing transcriptional regulators, influx and efflux transmembrane transporters, chaperones, and storage molecules. In this context, bacterial cells prevent Cu toxicity by expressing some of these molecules in response to high intracellular metal conditions. The cytoplasmic response to Cu^+^ excess has been characterized in numerous Gram-positive and Gram-negative bacteria (11, 16–19). Nevertheless, periplasmic components involved in Cu^+^ homeostasis have received much less attention. A simple consideration of the Gram-negative bacterium architecture points out that periplasmic dyshomeostasis is likely to precede the cytoplasmic response to a surge of Cu^+^ influx. Supporting this idea, mathematical simulations based on Cu^+^ uptake experiments in Pseudomonas aeruginosa under dyshomeostasis conditions suggest that the periplasmic Cu^+^ overload precedes the cytoplasmic imbalance (10). Moreover, periplasmic storage molecules are likely crucial for maintaining cellular Cu^+^ allocation (10).

Cytoplasmic Cu^+^-sensing transcriptional regulators are diverse, as different bacterial species have solved Cu^+^ homeostasis using alternative strategies (1, 5, 20, 21). However, the periplasmic response appears usually regulated by similar two-component systems (TCSs) (22, 23). Although absent in *Salmonella* (6), many *Enterobacteriaceae* (e.g., Escherichia coli, Klebsiella pneumoniae, etc.) modulate periplasmic Cu^+^ stress responses via the chromosomally encoded TCS CusRS and the plasmid-borne PcoRS (24–30). Instead, CopRS monitors extracytoplasmic Cu^+^ accumulation in Corynebacterium glutamicum and *Synechocystis* (31–33). CopRS is also found in *Pseudomonadaceae*, including Pseudomonas syringae (34, 35), P. aeruginosa (9), and Pseudomonas fluorescens (36, 37).

**FIG 1:**
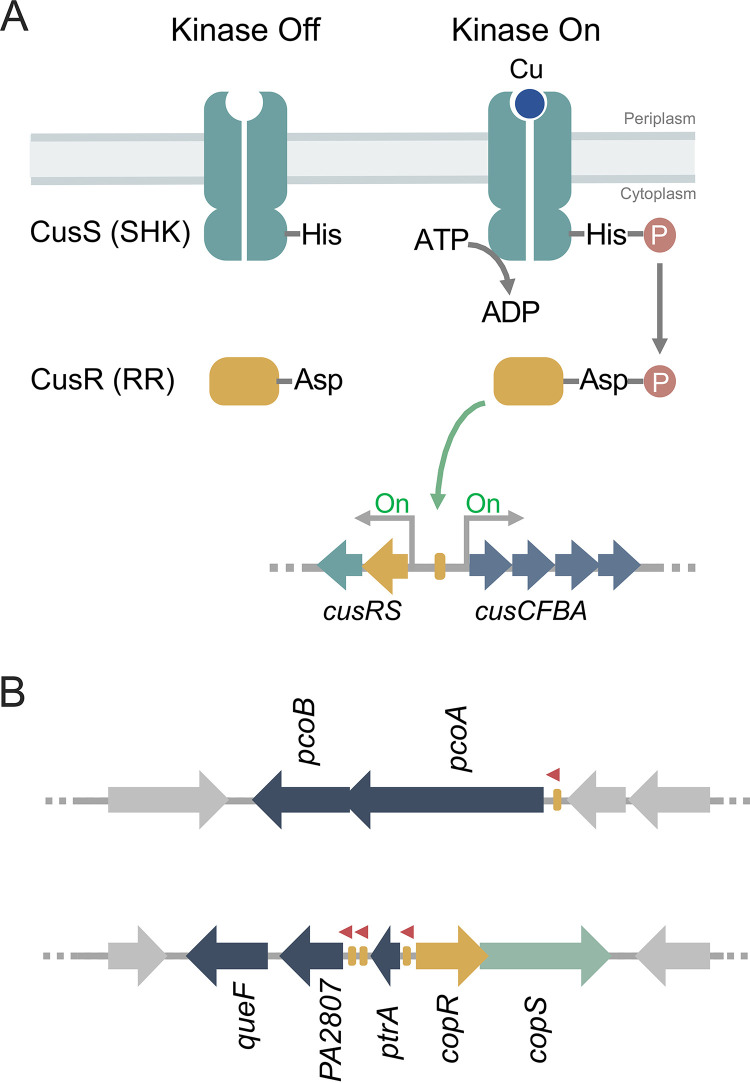
Transcriptional control mediated by TCSs. (A) Activation dynamics of canonical TCSs exemplified with the E. coli Cu-sensing CusRS. (B) Scheme of the TCS P. aeruginosa CopRS regulon. Promoter regions recognized by CopR (yellow rectangles) and transcription direction (red arrowheads) are shown. Overlapping arrows indicate that the start codon of second gene overlaps the stop codon of first gene in both *pcoAB* and *copRS* operons.

Most TCSs comprise a sensor histidine kinase (SHK) and its cognate cytoplasmic response regulator (RR). The SHK is usually a homodimeric membrane receptor with a periplasmic sensor domain flanked by two transmembrane segments (see Fig. S1 in the supplemental material). The C-terminal cytoplasmic domain contains the catalytic machinery (38). SHKs are bifunctional enzymes that switch between kinase and phosphatase states in a signal-dependent manner. In the kinase mode, the SHK undergoes autophosphorylation of a conserved His residue and subsequently transfers the phosphoryl group to a conserved Asp residue of its cognate RR. Although some RRs have alternative roles in their unphosphorylated states (39), phosphorylation of most of the RRs allosterically modifies their transcriptional activity (Fig. 1A). TCS sensors might also operate in a phosphatase mode. In these cases, the dephosphorylated SHK catalyzes the dephosphorylation of RR (RR∼P) that has been phosphorylated, metabolically or by an alternative kinase, in response to an environmental stimulus (39–43).

10.1128/mSphere.01193-20.1 FIG S1 Topology, functional domains, and location of transposon insertions in CopS. The periplasmic Cu^+^ sensor domain of CopS is highlighted (black solid line). His_41_ and His_140_ in blue and Phe_42_ in yellow are the residues forming the metal binding site. The C-terminal, cytoplasmic effector domain (black dotted line) contains the phosphorylatable His_235_ (red). Both insertional mutants, PW5705 and PW5706, have in-frame stop codons, producing shorter versions of CopS, lacking either part of the Cu binding residues and the effector domain (PW5705) or just the effector domain (PW5706). The CopS topology model was created using the Protter online tool version 1.0 (U. Omasits, C. H. Ahrens, S. Müller, and B. Wollscheid, Bioinformatics 30:884–886, 2014, https://doi.org/10.1093/bioinformatics/btt607). Download FIG S1, PDF file, 0.2 MB. Copyright © 2020 Novoa-Aponte et al. 2020 Novoa-Aponte et al. This content is distributed under the terms of the Creative Commons Attribution 4.0 International license.

Ultimately, the signal-dependent balance between SHK kinase and phosphatase activities determines the RR∼P levels, modulating the output response (38). In the archetypical E. coli CusRS TCS, Cu^+^ binding to the periplasmic loop of CusS promotes its autophosphorylation and the subsequent phosphorylation of the transcriptional regulator CusR (Fig. 1A). A positive regulation has then been assumed for TCS controlling periplasmic Cu^+^. Supporting this model, deletion of either the SHK CusS or the RR CusR leads to a reduced tolerance to external Cu^2+^, increased intracellular Cu^+^, and lack of transcriptional activation of regulated genes (e.g., *cusC*) (24–27).

The regulons controlled by the canonical Cu^+^-responsive TCS are limited to gene systems coding for the RNDs CusCFBA (26), PcoABCDRSE (27), and CopABCDRS (34, 35). However, Cu^+^ homeostatic pathways do not behave as evolutionary units. Instead, distinct species assemble different repertoires of metal handling proteins to achieve periplasmic Cu^+^ homeostasis (21). In particular, the P. aeruginosa CopRS regulon includes genes coding for an outer membrane transporter (PcoB), a multicopper oxidase (PcoA), and auxiliary proteins (PtrA, PA2807, and QueF) whose role in periplasmic Cu^+^ distribution is still unclear (44–46) (Fig. 1B). Interesting, the P. aeruginosa CusCBA transporter is not part of the CopRS regulon but is rather controlled by the cytoplasmic Cu^+^ sensor CueR (9). Given the distinct architecture of the P. aeruginosa CopRS regulon, a distinct sensing/activating mechanism for the control of periplasmic Cu^+^ homeostasis in *Pseudomonas* could be expected.

The structure of the isolated periplasmic domain of E. coli CusS shows four Ag^+^ (acting as Cu^+^ analog) binding sites per dimer (47). Two sites are symmetrically located at the dimer interface, and two are situated in outer loops of separated monomers. Reported estimates of metal-sensor affinities are limited and quite dissimilar among the different Cu-sensor histidine kinases. The E. coli CusS interacts with Ag^+^ with an affinity in the micromolar range (48), while *Synechocystis* CopS binds Cu^2+^ with high subattomolar affinity (32). Thus, significant aspects of sensor activation such as selectivity (Cu^+^ versus Cu^2+^) and sensitivity (affinity) are still undefined. These parameters will determine the level of free Cu in the periplasm and provide evidence for the metal redox status.

Here, we report that the transcriptional control of the CopRS regulon in P. aeruginosa relies on the Cu-dependent phosphatase activity of CopS, rather than on its kinase activity. Phosphorylation of the RR CopR and the consequent activation of the CopRS regulon appear independent of CopS. However, in the absence of Cu, CopS shuts down the transcriptional response to Cu^+^, likely dephosphorylating CopR. Then, when the periplasmic Cu^+^ level rises, the phosphatase activity of CopS is blocked, allowing the accumulation of phosphorylated CopR (CopR∼P) which promotes the expression of the periplasmic Cu^+^-homeostasis network. Finally, CopS binds both Cu^+^ and Cu^2+^ with similar high affinities, ensuring the absence of free Cu in the periplasm.

## RESULTS

CopRS controls P. aeruginosa periplasmic Cu^+^ homeostasis (9). Notably, there are significant differences between the CopRS regulon and those of other characterized Cu^+^-sensing TCSs, e.g., E. coli CusRS. The likely presence of additional mechanistic and molecular differences warranted a closer examination of CopRS function.

### Deletion of *copS* leads to Cu tolerance.

**FIG 2:**
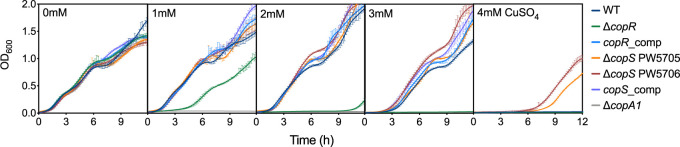
Cu tolerance of Δ*copR* and Δ*copS* mutant strains. Growth rate of WT, Δ*copR*, Δ*copS* (PW5705 and PW5706), Δ*copA1*, and CopR and CopS complemented strains in the absence or the presence of increasing (0 to 4 mM) concentrations of CuSO_4_. Data are the mean ± SEM from at least three independent experiments.

We initiated our studies by looking at the growth rate of Δ*copS* and Δ*copR* mutant strains in the presence of external Cu^2+^. Based on the mechanism of described Cu^+^-sensing TCSs (Fig. 1A), it was expected that the lack of either CopS or CopR would lower the cellular tolerance to external Cu^2+^. As anticipated, the Δ*copR* strain was more susceptible to Cu^2+^ than the wild-type (WT) strain (Fig. 2). In contrast, two independent *copS* transposon mutants, PW5705 and PW5706 (see Fig. S1 in the supplemental material), were surprisingly much more tolerant to external Cu^2+^ than the WT strain. As these phenotypes were reversed by complementation with the corresponding gene, all subsequent experiments were performed with the Δ*copS* PW5706 strain. For comparison, in addition to the WT strain, the well-characterized Cu^+^-sensitive Δ*copA1* mutant strain was included as a control in this initial phenotypical characterization (8).

**FIG 3:**
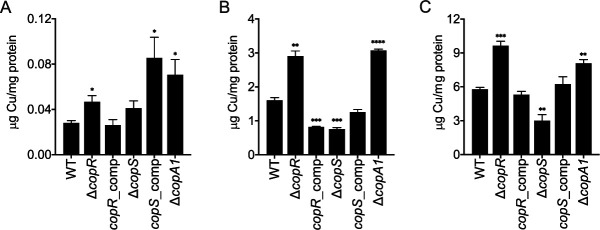
Whole-cell Cu levels in WT, Δ*copR*, Δ*copS*, Δ*copA1*, and CopR and CopS complemented strains under normal growth conditions (i.e., no additional CuSO_4_ added) (A) and after 10 min exposure to 2 mM CuSO_4_ (B) or 4 mM CuSO_4_ (C). Data are the mean ± SEM from three independent experiments. Significant differences from values with the WT strain as determined by unpaired two-tailed Student’s *t* test are *, *P* < 0.05; **, *P* < 0.01; ***, *P* < 0.001; ****, *P* < 0.0001.

Importantly, these growth phenotypes were the consequence of significantly different levels of intracellular Cu^+^ upon exposure to CuSO_4_ (Fig. 3). Thus, the Δ*copR* mutant strain accumulated more Cu^+^, while the Δ*copS* cells stored less metal, than the WT strain. Again, alterations in Cu^+^ levels were reversed by gene complementation of the mutant strains. These differences in Cu tolerance and cellular metal levels observed for the Δ*copR* and Δ*copS* mutant strains cannot be explained by the currently accepted model derived from the E. coli TCS CusRS (Fig. 1A) and suggest an alternative mechanism for coupling periplasmic Cu^+^ sensing and gene expression in P. aeruginosa.

### The CopRS regulon is expressed in the Δ*copS* mutant strain independently of the Cu^+^ levels.

**FIG 4:**
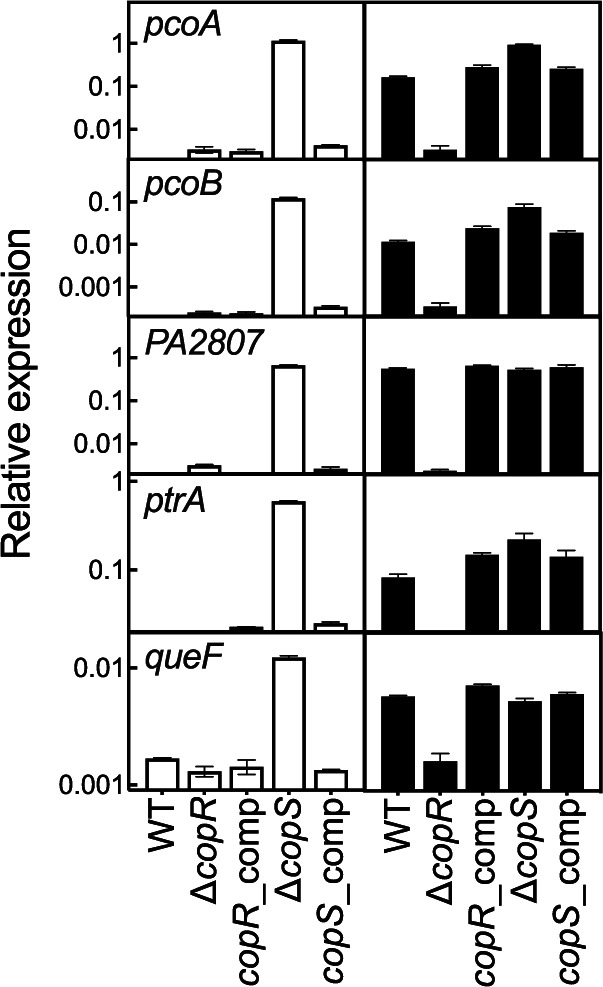
Expression of genes in the CopRS regulon in WT, Δ*copR*, Δ*copS*, and corresponding complemented strains in the absence (white) and the presence (black) of 0.5 mM CuSO_4_ (5-min treatment). Transcript levels of *pcoA*, *pcoB*, *PA2807*, *ptrA*, and *queF* genes are plotted relative to that of the housekeeping gene *PA4268*. Data are the mean ± SEM from three independent experiments.

Toward understanding the increased Cu tolerance and intracellular levels in the Δ*copS* strain, we investigated the transcriptional response to Cu^2+^ exposure of the CopRS regulon in the Δ*copR* and Δ*copS* mutant strains. We have described that CopRS controls the expression of *pcoA*, *pcoB*, *ptrA*, *queF*, and *PA2807* coding for periplasmic and outer membrane proteins (Fig. 1B) (9). As previously observed in the WT strain, genes of the CopRS regulon are induced in response to external Cu^2+^ exposure (Fig. 4). As expected, their Cu-induced expression was abolished in the Δ*copR* mutant. In contrast, the Δ*copS* mutant strain showed a constitutive activation of all the genes of the CopRS regulon, even in the absence of the Cu^2+^ stimulus. In the Δ*copS* background, expression of these genes was maximal and independent of the presence of Cu^2+^ in the culture medium. That similar expression pattern of the CopRS-activated genes in the Δ*copS* strain was attained in the absence of added Cu^2+^ and in the presence of low, nondeleterious Cu^2+^ levels (0.5 mM), intermediate toxic Cu^2+^ levels (2 mM), and lethal Cu^2+^ levels (4 mM) (Fig. S2). This suggests that CopS is not required to activate, i.e., phosphorylate, CopR. The activation of CopR in the Δ*copS* mutant in the absence of supplemented Cu^2+^ points to a mechanism where the phosphatase activity of CopS maintains low levels of CopR∼P under noninducing conditions. The Δ*copS* strain failure to maintain the system *off* in the absence of added Cu was reversed in the complemented strain (Fig. 4). The transcriptional analyses also showed that the expression of the *copRS* operon is not autoregulated (Fig. S3). That is, even though *copRS* expression is induced in response to Cu^+^, it was not affected either in the Δ*copR* or in the Δ*copS* mutant strain. Noticeably, the repressed transcription of *oprC*, coding for the outer membrane Cu importer (9, 49), was further repressed in the Δ*copS* mutant strain, consistent with the Cu^+^-tolerant phenotype, i.e., less intracellular Cu, exhibited by this strain (Fig. S4A). Conversely, the increased transcription of genes in the CueR regulon (*copA1* and *cusA*) in response to Cu^+^ was not altered either in the Δ*copR* or in the Δ*copS* mutant strain (Fig. S4B). This confirms that the lack of transcriptional control observed in the Δ*copR* and Δ*copS* mutant strains is limited to the genes of the CopRS regulon. Maximal transcription of the CopRS-activated genes in the Δ*copS* strain, even in the absence of external Cu^2+^ stress, requires CopR∼P. As mentioned before, RRs can be phosphorylated either by alternative kinases or metabolically, by physiologically relevant small phosphodonors like the acetyl phosphate pool (39–43). This pool, in turn, depends on the activity of two enzymes, the phosphate acetyltransferase Pta and the acetate kinase AckA. Testing the role of acetyl phosphate on CopR phosphorylation, the Cu^2+^ resistance of the Δ*pta* and Δ*ackA* strains was evaluated (Fig. S5). Both strains showed a Cu^2+^ sensitivity profile similar to that of the WT strain, suggesting that phosphorylation of CopR does not depend on the acetyl phosphate pool and the involvement of a yet-unidentified SHK.

10.1128/mSphere.01193-20.2 FIG S2 Expression of genes in the CopRS regulon in the Δ*copR* and Δ*copS* mutant strains quantified in the absence (white) and the presence (black) of 0.5, 2, and 4 mM CuSO_4_ (5-min treatment). Transcript levels of *pcoA*, *pcoB*, *PA2807*, *ptrA*, and *queF* genes are plotted relative to that of the housekeeping gene *PA4268*. Data are the mean ± SEM from three independent experiments. Download FIG S2, PDF file, 0.04 MB. Copyright © 2020 Novoa-Aponte et al. 2020 Novoa-Aponte et al. This content is distributed under the terms of the Creative Commons Attribution 4.0 International license.

10.1128/mSphere.01193-20.3 FIG S3 Expression of the *copRS* operon in the Δ*copR* and Δ*copS* mutant strains quantified in the absence (white) and the presence (black) of 0.5, 2, and 4 mM CuSO_4_ (5-min treatment). Transcript levels of *copR* and *copS* genes are plotted relative to that of the housekeeping gene *PA4268*. Data are the mean ± SEM from three independent experiments. Download FIG S3, PDF file, 0.03 MB. Copyright © 2020 Novoa-Aponte et al. 2020 Novoa-Aponte et al. This content is distributed under the terms of the Creative Commons Attribution 4.0 International license.

10.1128/mSphere.01193-20.4 FIG S4 Expression of Cu transporter genes in the Δ*copR* and Δ*copS* mutant strains quantified in the absence (white) and the presence (black) of 0.5, 2, and 4 mM CuSO_4_ (5-min treatment) (11). Transcript levels of *copA1*, coding for the Cu^+^ efflux P_1B_-type ATPase CopA1; *cusA*, a component of the RND CusABC system (A); and *oprC*, coding for Cu importer OprC (B), are plotted relative to that of the housekeeping gene *PA4268*. Data are the mean ± SEM from three independent experiments. Download FIG S4, PDF file, 0.04 MB. Copyright © 2020 Novoa-Aponte et al. 2020 Novoa-Aponte et al. This content is distributed under the terms of the Creative Commons Attribution 4.0 International license.

10.1128/mSphere.01193-20.5 FIG S5 Cu tolerance of Δ*ackA* and Δ*pta* mutant strains. Growth rate of WT, Δ*copR*, Δ*copS*, Δ*ackA*, and Δ*pta* strains in the presence of 0 to 4 mM CuSO_4_. Data are the mean ± SEM from at least three independent experiments. Download FIG S5, PDF file, 0.1 MB. Copyright © 2020 Novoa-Aponte et al. 2020 Novoa-Aponte et al. This content is distributed under the terms of the Creative Commons Attribution 4.0 International license.

### His_235_ acts as a switch to turn on/off the CopS signaling pathway.

The cytoplasmic region of the SHK sensory proteins contains the catalytic domain and the phosphotransfer domain able to switch between kinase and phosphatase activities in a signal-dependent manner (42, 50). In most SHKs, this phosphotransfer domain contains an invariant His residue that autophosphorylates in the first step of the signaling cascade, activating the kinase state of the SHK. Subsequently, the RR protein is phosphorylated in a highly conserved phosphoacceptor Asp, leading to the transcriptional induction of its activated genes (51) (Fig. 1A). In contrast to the kinase state, in the phosphatase state a dephosphorylated SHK removes the phosphate group from the RR (42). The kinase and phosphatase states are mutually exclusive. In some cases, the activation of the kinase state is associated with phosphatase deactivation with the consequent accumulation of phosphorylated RR. The observed phenotypes in Δ*copS* and Δ*copR* strains suggest that in the absence of Cu^+^, CopS acts as a phosphatase dephosphorylating CopR∼P. Then, when CopS senses Cu^+^, its phosphatase would be inactivated, leading to a rise of CopR∼P, triggering the expression of the CopRS regulon. Testing these ideas, the phosphorylatable residues, His_235_ in CopS and Asp_51_ in CopR, were identified by sequence alignment with characterized TCS (Fig. S6). Site-directed mutagenesis was performed to generate Asp_51_Ala and Asp_51_Glu replacements in CopR and His_235_Ala in CopS coding sequences, and the resulting constructs were employed to complement the corresponding Δ*copR* and Δ*copS* mutant strains.

10.1128/mSphere.01193-20.6 FIG S6 Multiple sequence alignment of the P. aeruginosa CopRS TCS proteins with bacterial homologs. (A) P. aeruginosa CopR protein sequence was aligned with characterized bacterial RR to identify the conserved phosphorylatable Asp residue (highlighted in blue). (B) P. aeruginosa CopS and E. coli CusS protein sequences were aligned with homologs of both of CopS-like and CusS-like proteins from different species. Conserved Cu binding sites at the dimeric interface are highlighted in yellow. E. coli CusS Cu binding sites, not conserved in CopS, are highlighted in orange. The conserved phosphorylatable His residue is highlighted in blue. UniProt accession numbers precede each species name. Download FIG S6, PDF file, 0.1 MB. Copyright © 2020 Novoa-Aponte et al. 2020 Novoa-Aponte et al. This content is distributed under the terms of the Creative Commons Attribution 4.0 International license.

**FIG 5:**
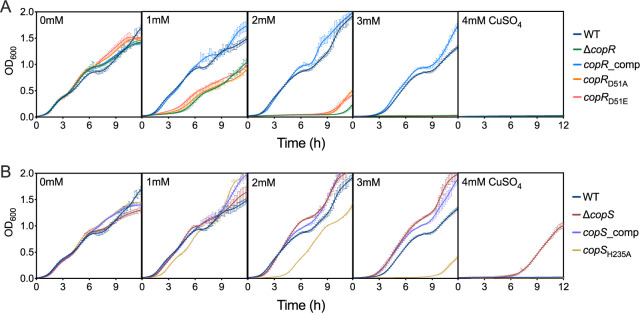
Cu tolerance of Δ*copR* and Δ*copS* mutant strains complemented with CopR and CopS mutant proteins lacking the phosphorylatable residues. (A) Growth rate of the Δ*copR* mutant complemented with *copR*_D51A_ or *copR*_D51E_ in the absence or the presence of increasing (0 to 4 mM) concentrations of CuSO_4_. (B) Growth rate of the Δ*copS* mutant complemented with *copS*_H235A_ in the presence of 0 to 4 mM CuSO_4_. Data are the mean ± SEM from three independent experiments.

**FIG 6:**
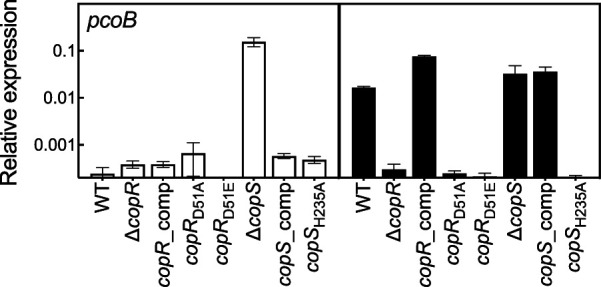
Expression of *pcoB* in Δ*copR* and Δ*copS* mutant strains complemented with CopR and CopS lacking the phosphorylatable residues. *pcoB* expression was determined in the absence (white) and the presence (black) of 2 mM CuSO_4_ (5-min treatment) in the indicated strains. The Δ*copR* mutant was complemented with *copR* coding for substitutions Asp_51_Ala and Asp_51_Glu. The Δ*copS* mutant was complemented with the *copS* gene coding for substitution His_235_Ala. Transcript levels of *pcoB* are plotted relative to the housekeeping gene *PA4268*. Data are the mean ± SEM from three independent experiments.

Figure 5A shows that the mutations Asp_51_Ala and Asp_51_Glu in CopR lead to growth phenotypes comparable to that of the Δ*copR* strain. This pointed to the requirement of Asp at this position for CopR function and revealed that the Glu residue does not act as a phosphomimetic residue. In agreement, Fig. 6 shows that neither CopR_D51A_ nor CopR_D51E_ was able to activate *pcoB* expression in the presence of external Cu^2+^, a lack of function associated with the absence of the Asp_51_ phosphorylation. Conversely, the His_235_Ala CopS mutant behaved differently from both the WT and the Δ*copS* strain. In contrast to the Cu^2+^ tolerance phenotype observed for the Δ*copS* mutant, the His_235_Ala CopS mutant had an increased sensitivity to Cu^2+^ (Fig. 5B), suggesting that the phosphatase activity of CopS remains functional in the absence of His_235_. Analysis of the transcriptional activation of genes in the CopRS regulon further supports this idea. In the absence of supplemented Cu^2+^, *pcoB* transcription remained low in the His_235_Ala CopS mutant, similar to the level observed in the WT strain and in contrast to the increased expression in the Δ*copS* mutant strain. In fact, addition of external Cu^2+^ did not promote the transcription of *pcoB* in the His_235_Ala mutant, similar to the *pcoB* expression pattern in the Δ*copR* strain and clearly different from the induction observed in the WT and the maximal expression attained in the Δ*copS* mutant. The more marked *pcoB* expression defect under Cu stress of the *copS*_H235A_ strain compared to the Δ*copR* strain is likely associated with experimental conditions. Importantly, the lack of transcriptional activation of *pcoB* suggests that the His_235_Ala CopS mutant was not able to respond to changes in periplasmic Cu^+^ levels, explaining the Cu^2+^-sensitive phenotype observed for this strain (Fig. 5B) and suggesting that the His_235_Ala mutation locked CopS in a phosphatase-ON state irresponsive to the presence of Cu.

### CopS periplasmic sensor domain binds two Cu^+^ ions per functional unit.

Most TCS sensors are homodimeric membrane proteins. The periplasmic sensor domain of CopS, flanked by two transmembrane segments (Fig. S1), extends between residues 34 and 151 [CopS_(34–151)_]. The function of the system relies on its ability to bind cognate metal ions. To explore CopS metal binding properties, the P. aeruginosa CopS_(34–151)_ sensor domain carrying alternative His or Strep tags was heterologously expressed and purified to homogeneity (Fig. S7). His-tagged proteins were used in Cu^+^ binding, while the Strep-tagged fragments were used in Cu^2+^ binding experiments.

10.1128/mSphere.01193-20.7 FIG S7 SDS-PAGE analysis of the periplasmic copper binding loop of CopS_(34–151)_. Ten micrograms of purified His_6_-tagged (A) or Strep-tagged (B) protein was subjected to 8 to 16% gradient SDS-PAGE. Gels were stained with Coomassie blue G250. Left lanes: molecular weight marker. Right lanes: purified proteins. Arrows indicate the protein monomers and dimers, with expected masses of 19 and 38 kDa, respectively. The presence of the C-terminal His_6_ tag in CopS_(34–151)_ stabilized the dimer form of the protein. The C-terminal Strep tag did not. The gel shown in panel A was spliced for labeling purposes (blue vertical line). Download FIG S7, PDF file, 1.1 MB. Copyright © 2020 Novoa-Aponte et al. 2020 Novoa-Aponte et al. This content is distributed under the terms of the Creative Commons Attribution 4.0 International license.

**FIG 7:**
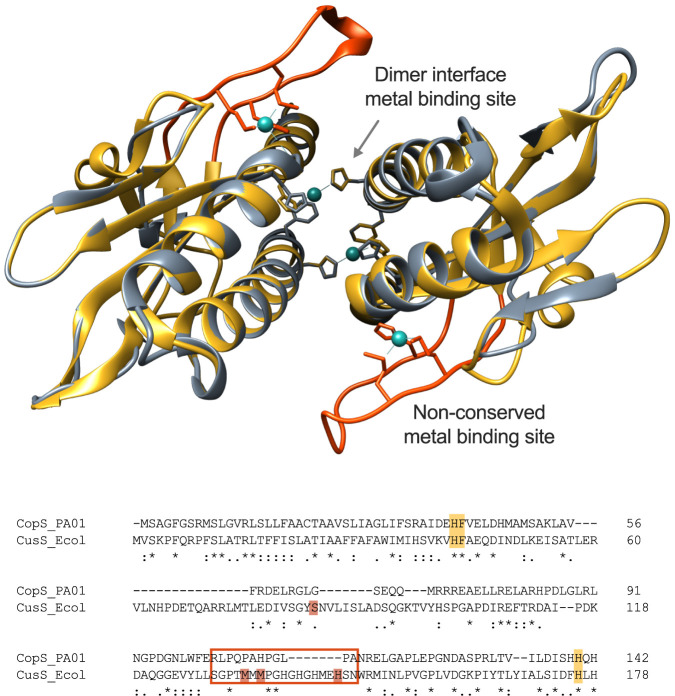
Structural superposition of the periplasmic Cu^+^ binding loop of P. aeruginosa CopS (gray) and E. coli CusS (yellow). The structure of CopS was modeled using the CusS structure as the template (PDB ID: 5KU5 [43]). An overall root mean square deviation of 0.791 Å (Cα atoms) was calculated for the superposition of CopS and CusS structures. Conserved Cu binding sites at the dimeric interface (His_41_, Phe_42_, and His_140_) are shown as sticks in the structural model and highlighted in yellow in the sequence alignment. The Cu^+^ binding sites within the CusS orange loops (framed in rectangle in the alignment) are not conserved in CopS.

The Cu^+^ binding stoichiometry of the isolated domain was first measured at a saturating metal concentration (five times molar excess) in the presence of dithiothreitol (DTT) as reducing agent. The CopS_(34–151)_ dimer was able to bind 2.3 ± 0.5 Cu^+^. This differs from the stoichiometry of four Ag^+^ (used as Cu^+^ analog) per dimer observed in E. coli CusS (47). However, the periplasmic sensor domain of CopS homolog proteins is considerably shorter than the CusS domain, lacking a loop containing residues (Ser_84_, Met_133_, Met_135_, and His_145_) involved in metal binding in CusS (Fig. S6B). In effect, a phylogenetic tree built with sequences homologous to CopS and CusS (>45% identity) shows a clear evolution of two distinct subgroups of CusS homologs in *Enterobacterales* and in *Burkholderiales* and a separate group of CopS homologs in *Pseudomonadales* (Fig. S8). This structural difference leading to the alternative stoichiometry can be more easily observed when the homology modeling of P. aeruginosa CopS is overlapped with the crystal structure of the Ag^+^-bound periplasmic sensor domain of E. coli CusS (47) (Fig. 7). The two symmetric metal binding sites fully conserved in both CopS and CusS are located at the dimeric interface. Each site is formed by two invariant His residues (His_41_ and His_140_ in CopS), one from each dimer subunit. A Phe residue likely interacting with the metal in CusS is also conserved in CopS (Phe_42_). These are probably the Cu^+^-sensing sites involved in signal transduction. On the other hand, the structural comparison clearly shows that the loop containing the additional metal binding sites of CusS is missing in CopS (orange loops, Fig. 7).

10.1128/mSphere.01193-20.8 FIG S8 Phylogenetic tree of CopS-like and CusS-like proteins. Separately, P. aeruginosa CopS and E. coli CusS were used to find homologs in the UniProtKB database. The top 20 hits (>45% homology) from each BLAST search were aligned with Clustal Omega, and the resulting alignment was used to construct the displayed average distance tree. Different taxa were colored as follows: dark blue, *Enterobacterales*; cyan, *Burkholderiales*; and pale violet, *Pseudomonadales*. UniProt accession numbers precede each species name. Download FIG S8, PDF file, 0.1 MB. Copyright © 2020 Novoa-Aponte et al. 2020 Novoa-Aponte et al. This content is distributed under the terms of the Creative Commons Attribution 4.0 International license.

### The CopS periplasmic sensor binds Cu ions with femtomolar affinities.

**FIG 8:**
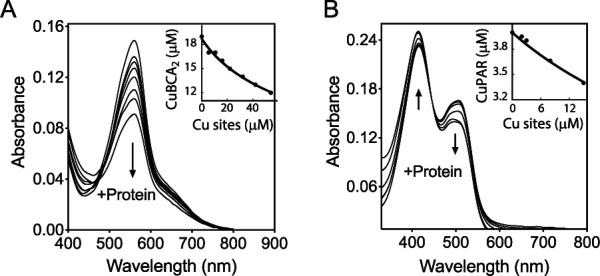
Determination of the dissociation constants *K_D_* of the periplasmic Cu binding loop of CopS_(34–151)_. (A) Spectrophotometric titration of 100 μM BCA and 18.7 μM Cu^+^ with 10 to 50 μM His-tagged CopS_(34–151)_. The arrow indicates the decrease in absorbance at 562 nm upon protein addition. The inset shows the fitting of the data set to equation 2 with a *K_D_* of (2.77 ± 0.07) × 10^−14^ M (*R*^2^ 0.992). Two Cu sites per CopS dimer are assumed. (B) Spectrophotometric titration of 10 μM PAR and 4 μM Cu^2+^ with 2 to 20 μM Strep-tagged CopS_(34–151)_. The arrows indicate the increase in absorbance at 415 nm and the decrease at 562 nm upon protein addition. The inset shows the fitting of the data set to equation 4 with a *K_D_* of (3.3 ± 0.1) × 10^−14^ M (*R*^2^ 0.984).

By analogy with how cytoplasmic sensor metal affinities are tuned to maintain free metal levels (52, 53), the affinity of CopS for Cu^+^ ions will certainly have determinant effects on free (hydrated) Cu^+^ ion levels in the periplasm. Exploring the binding of Cu^+^ to CopS, we measured the sensor metal binding affinity using competing ligands. The ligands were present in excess to ensure effective competition. In all cases, the determinations were performed assuming that both Cu sites at the CopS dimer interface were functionally independent and thermodynamically indistinguishable. Initial determinations of CopS_(34–151)_ affinity for Cu^+^ using bathocuproine disulfonate (BCS) as a competitor showed a limited but measurable decrease in the absorbance of the [Cu^I^(BCS)_2_^3−^] complex, corresponding to a *K_D_* (dissociation constant) value of CopS_(34–151)_ for Cu^+^ of 2.2 × 10^−14^ M (data not shown). However, it was apparent that CopS was not an effective competitor with BCS for the metal. Instead, 2,2′-bicinchoninic acid (BCA), with a lower affinity for copper than that of BCS, appeared more appropriate to measure affinities in the femtomolar range (54). Using BCA as the competing ligand and fitting titration curves to equation 2, a CopS_(34–151)_-Cu^+^ *K_D_* of (2.77 ± 0.07) × 10^−14^ M was obtained (Fig. 8A). This appears within the range of affinities observed for many other Cu^+^ binding molecules (11, 54, 55).

*Synechocystis* CopS binds Cu^2+^ with high subattomolar affinity (Cu^+^ binding stoichiometry was not reported) (32). Exploring the possibility of high-affinity Cu^2+^ binding to P. aeruginosa CopS, the chromogenic ligand 4-(2-pyridylazo)resorcinol (PAR) was used as a competitive ligand for Cu^2+^ with purified Strep-tagged CopS_(34–151)_ (Fig. S7), in the absence of reducing agents. A CopS_(34–151)_-Cu^2+^ *K_D_* of (3.3 ± 0.1) × 10^−14^ M was observed (Fig. 8B). Consequently, it is apparent that CopS_(34–151)_ binds both Cu^+^ and Cu^2+^ with quite similar affinities in the femtomolar range. These high affinities provide insights into the *in vivo* metal dynamics and virtual absence of free Cu ions in the bacterial periplasm.

## DISCUSSION

The relevance of the periplasmic Cu pool in the P. aeruginosa response to Cu^2+^ stress is well established (10, 56). Results presented here show novel important characteristics of the P. aeruginosa TCS CopRS. The sensor has a negative-control mechanism based on its phosphatase rather than on its kinase activity. At the dimer interface, it binds two Cu^+/2+^ ions with femtomolar affinities, likely resulting in the absence of periplasmic free Cu. This CopRS distinct Cu^+^ signaling mechanism is in line with the other unique features of the P. aeruginosa Cu homeostasis network, namely, cytoplasmic and periplasmic sensors with singular regulons, an RND-transporter regulated by the cytoplasmic sensor, and multiple cytoplasmic Cu^+^ chaperones and efflux P_1B_-ATPases (8–11, 57). The emerging model challenges a number of ideas associated with early studies of the E. coli CusRS TCS. Along with *Salmonella*, which has distinct Cu^+^ balance mechanisms (6), P. aeruginosa provides a clear example of alternative approaches used by bacteria to achieve Cu homeostasis.

### CopS Cu-dependent phosphatase activity mediates signal transduction.

Characterization of CopRS was initiated by analyzing the tolerance of Δ*copS* and Δ*copR* strains to external Cu^2+^. While an increased sensitivity was expected based on the reported phenotypes of E. coli Δ*cusS* and Δc*usR* strains, the Δ*copS* strain showed higher tolerance to external Cu^2+^. Although unexpected, this phenomenon has been previously observed, albeit unnoticed. It was reported that deletion of the P. aeruginosa CopS did not compromise the ability of the bacteria to grow in the presence of Cu^2+^ (58). Furthermore, there was no evident Cu-induced expression of a *lacZ* transcriptional fusion to a Pseudomonas putida CinRS (a CopRS ortholog)-dependent promoter in a P. aeruginosa Δ*copR* background. However, Cu-independent expression of the same reporter was attained in the P. aeruginosa Δ*copS* background (59). Also similar to the P. aeruginosa Δ*copS* strain, a P. fluorescens Δ*copS* strain was more tolerant to external Cu^2+^ (36).

**FIG 9:**
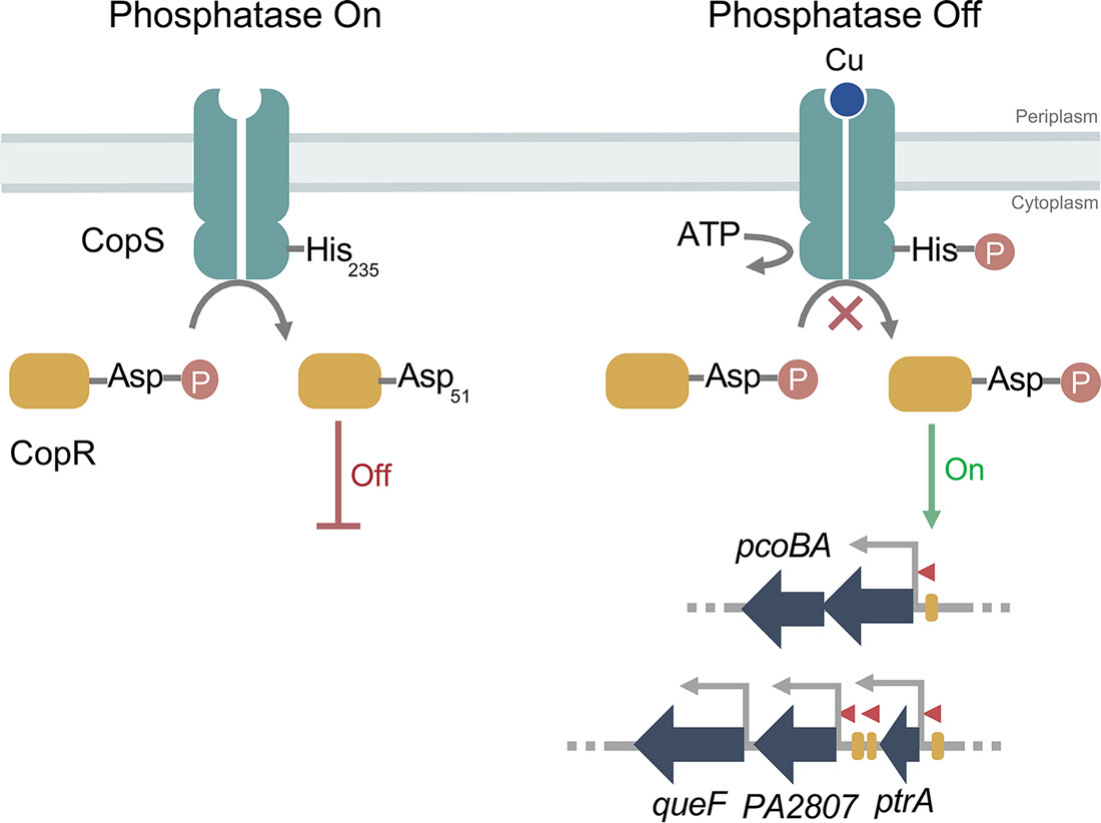
Model of the phosphatase-based mechanism of the P. aeruginosa CopRS. Phosphatase On: when periplasmic free Cu remains under the subfemtomolar level, the CopS phosphatase activity maintains low levels of phosphorylated CopR, shutting *off* the transcriptional response to high periplasmic Cu. Phosphatase Off: upon Cu binding, CopS autophosphorylates at His_235_. This turns *off* the CopS phosphatase activity, allowing the accumulation of phosphorylated CopR and triggering the expression of the CopRS regulon (i.e., *pcoA*, *pcoB*, *queF*, *PA2807*, and *ptrA*).

The Cu^2+^ resistance phenotype of the P. aeruginosa Δ*copS* strain is supported by the maximal expression of the CopRS regulon and the consequent reduced whole-cell Cu^+^ content. The simplest explanation for these observations is a mechanism where, in the absence of Cu, the CopS phosphatase activity abrogates the induction of the CopRS regulon by maintaining low levels of CopR∼P (Fig. 9). When CopS detects periplasmic Cu overload, its phosphatase activity is blocked allowing the accumulation of CopR∼P, which promotes the expression of the periplasmic Cu homeostasis network.

Signal transduction by archetypical TCSs relies on bifunctional kinase/phosphatase SHKs (60). A positive action results from sensor autokinase activity and phosphotransfer to the RR while negative regulation involves the sensor phosphatase activity (50). The ultimate determining factor of the cascade activation is the phosphorylation status of the RR. Accumulation of RR∼P is the consequence of a signal-dependent stimulation of the sensor-kinase activity or a signal-dependent blockage of the sensor-phosphatase activity. While we cannot rule out the absence of autokinase activity, or sensor phosphorylation by an alternative kinase, the most parsimonious model to explain our data is that CopS, under our experimental conditions, harbors autokinase and phosphatase activities. The signal-independent activation of the CopRS regulon in the Δ*copS* background evidences the requirement of the CopS phosphatase activity to maintain low levels of CopR∼P in the absence of Cu. It is also apparent that CopS is not required for the phosphorylation of CopR, implying that an alternative mechanism for the phosphorylation of CopR should exist. There is extensive evidence that RRs can be phosphorylated (cross-phosphorylated) by endogenous phosphodonors (39, 41–43). In the case of CopR, acetyl phosphate does not seem to be the donor. Alternative mechanisms for RR phosphorylation known as many-to-one or one-to-many, where many SHKs phosphorylate a given RR or a single SHK phosphorylates multiple RRs, have been proposed (38, 60). It could then be argued that CopR phosphorylation might be the consequence of an unspecific cross talk with a noncognate SHK that occurs only in the absence of CopS. However, such cross talk has been observed only when both the reciprocal RR and the cognate SHK were absent (41). These conditions are distinct from those in our experiments.

The evidence indicates that Cu-dependent CopS autokinase activity, or at least the integrity of His_235_, is required for the inhibition of the CopS-phosphatase activity. His_235_Ala replacement leads to a Cu^+^-independent inactivation of the regulon, suggesting a constitutively active phosphatase activity. While this points out that His_235_ is not required for the CopS phosphatase activity, it implies that Cu-dependent CopS autophosphorylation turns *off* the CopS phosphatase activity, leading to accumulation of CopR∼P. That is, as described previously, the dephosphorylated SHKs have phosphatase activity (42, 50).

### CopS binds two Cu ions with femtomolar affinities.

Its Cu binding characteristics are what defines the function of CopS. We determined that P. aeruginosa CopS binds two metal ions with an affinity in the 3 × 10^−14^ M range. Little information is available regarding the binding stoichiometry and affinities of other Cu-sensing TCS sensors. The structure of E. coli CusS clearly supports a stoichiometry of four metal atoms per CusS-sensing dimer (47). Two of these ions bind at the dimer interface, while the other two attach to external loops, one in each subunit. Structural comparison of P. aeruginosa CopS and E. coli CusS shows that both types of sensors would bind and sense the metal with conserved His residues at the dimer interface. However, the CusS extra sites are not conserved in CopS or in its homologs. Regarding binding affinities, E. coli CusS binds Ag^+^ with a reported 8 μM affinity, measured in equilibrium dialysis experiments (48); in contrast, *Synechocystis* CopS binds Cu^2+^ with subattomolar affinity (32). It would be quite speculative to compare such dissimilar determinations. However, it might be instructive to consider the observed 10^−19^ to 10^−21^ M affinities of cytoplasmic copper sensors in general (55, 61) and those determined for the cytoplasmic triad CopZ2/CueR/CopZ1 of P. aeruginosa, with relative affinities for Cu^+^ ranging between 10^−15^ and 10^−17^ M (9, 11). The weaker affinity of CopS than of the cytoplasmic regulators and chaperones is likely the consequence of a metal binding site formed by His rather than Cys residues. This is a logical arrangement, given the possible oxidation of proximal Cys under periplasmic redox stress. Importantly, a femtomolar affinity still supports the idea that there would not be free Cu^+/2+^ in the cell periplasm, as shown for the cytoplasm (55, 62). However, the relative binding strength of CopS is likely to be linked to those of periplasmic Cu^+^ chaperones that exchange metal with the sensor. That is, the proteins should be able to exchange the metal. However, as shown with cytoplasmic chaperone/sensor partners, the protein-protein binding affinity will have a significant effect in the final exchange constant (11).

CopS binds both Cu^+^ and Cu^2+^ with similar high affinities. It is accepted that cytoplasmic transporters and chaperones bind and distribute cuprous ions. However, the periplasm is a more oxidizing compartment (63, 64), containing enzymes such as the multicopper oxidase PcoA present in the periplasm of P. aeruginosa (65). It has been proposed that periplasmic enzymes might catalyze Cu^+^ oxidation to the assumed less toxic Cu^2+^ (66). However, free (hydrated) Cu^+^ would be spontaneously oxidized by O_2_ in an aerobic environment. Then, the redox status of periplasmic Cu is unclear and beyond the goals of this report. We presume that Cu oxidation state will depend on the molecule interacting with and delivering Cu to CopS. In any case, the capability to bind both Cu^+^ and Cu^2+^ might help CopS to sense the metal under redox stress.

### The distinct CopRS mechanism is in line with the singular architecture of the P. aeruginosa Cu homeostasis system.

E. coli and *Salmonella* are the frequent models to explore transition metal homeostasis in Gram-negative bacteria. However, recent studies of P. aeruginosa have begun to show different novel molecular strategies to sense, buffer, and distribute Cu^+^ (8–10, 67). For instance, consider how the regulons of both compartmental sensors, CopRS and CueR, differ among these three organisms (6, 9, 24, 68, 69). Also, analyze the multiple functionally distinct homologous Cu^+^ ATPases present in *Salmonella* and *Pseudomonas* and how these three Gram-negative bacteria have solved cytoplasmic Cu^+^-chaperoning using alternative strategies (6, 11, 70). Along with these observations, the relevance of periplasmic Cu^+^ sensing, storage, and transport has become more apparent. Then, it is not surprising that these model systems solve periplasmic Cu^+^ sensing either via a kinase sensor (CusRS, E. coli), an integration of a cytoplasmic Cu sensor with a general envelope stress response TCS (CueR-CpxRS, *Salmonella* [71]) or a phosphatase sensor (CopRS, P. aeruginosa). The evolutive and ecological advantages of these systems are still to be discovered and will be the subject of future enquiries in the field.

## MATERIALS AND METHODS

### Bacterial strains, plasmids, and growth conditions.

Bacterial strains, plasmids, and primers used in this study are listed in Table S1 in the supplemental material. P. aeruginosa PAO1 served as WT strain. Mutant strains PW5704 (Δ*copR*), PW5705 (Δ*copS*), PW5706 (Δ*copS*), PW2519 (Δ*pta*), and PW2520 (Δ*ackA*) were obtained from the P. aeruginosa PAO1 transposon mutant library (University of Washington, Seattle, WA) (72, 73). P. aeruginosa strains were grown at 37°C in Luria-Bertani (LB) medium supplemented with 25 μg/ml Irgasan, 30 μg/ml tetracycline (mutant strains), or 30 μg/ml gentamicin (complemented strains). E. coli strains were grown at 37°C in LB medium supplemented with 100 μg/ml ampicillin, 30 μg/ml kanamycin, or 10 μg/ml gentamicin, depending on the plasmid selection.

10.1128/mSphere.01193-20.9 TABLE S1 Bacterial strains, plasmids, and primers used in this study. Download Table S1, PDF file, 0.1 MB. Copyright © 2020 Novoa-Aponte et al. 2020 Novoa-Aponte et al. This content is distributed under the terms of the Creative Commons Attribution 4.0 International license.

### Construction of P. aeruginosa complemented strains.

Mutant strains were complemented with the corresponding gene under the control of the native promoter using the mini-Tn7T insertion system (74). Briefly, the genes and their 500-bp upstream promoter regions were amplified by PCR. The 3′ primer included a His_6_ tag coding sequence. Amplicons were cloned into the pUC18-mini-Tn7T-Gm suicide delivery vector. These plasmids were used as the template to introduce mutations coding for single substitutions *copR*_D51A_, *copR*_D51E_, and *copS*_H235A_ using Gibson assembly (75). The resulting plasmids were then introduced into recipient strains by conjugation, using the helper strains SM10(λpir)/pTNS2 and HB101/pRK2013. Conjugants were selected on 30 μg/ml gentamicin-25-μg/ml Irgasan-LB plates. Complemented strains were verified by PCR.

### Cu^2+^ sensitivity assay.

Overnight cultures were diluted in 25 μg/ml Irgasan-LB medium, adjusted to an optical density at 600 nm (OD_600_) of 0.05, and supplemented with the indicated CuSO_4_ concentration. Cell growth in 0.2 ml liquid medium was monitored for 24 h (OD_600_) at 37°C with continuous shaking using an Epoch 2 microplate spectrophotometer (BioTek).

### Whole-cell Cu content.

Cells (mid-log phase) were incubated in LB medium supplemented with 0.5, 2, or 4 mM CuSO_4_. Aliquots were taken after 10 min, treated with two times molar excesses of DTT and BCS, and harvested by centrifugation at 17,000 × *g*, 1 min. Pellets were washed twice with 150 mM NaCl and mineralized with fuming HNO_3_ (trace metal grade) for 60 min at 80°C and 2 M H_2_O_2_ for 60 min at room temperature. Cu levels were measured using atomic absorption spectroscopy (AAS) as described previously (9).

### Gene expression analysis.

Cells (mid-log phase) were incubated in antibiotic-free LB medium supplemented with 0.5, 2, or 4 mM CuSO_4_. In all cases, 0.5-ml aliquots were taken at 5 min and stabilized with RNAprotect bacterial reagent (Qiagen), and RNA was isolated with the RNeasy minikit (Qiagen). RNA was treated with DNase I, purified by phenol-chloroform extraction, and ethanol precipitated. One microgram of RNA was used for cDNA synthesis using the ProtoScript II kit (New England BioLabs). qPCRs were carried out with FastStart Essential DNA Green Master (Roche) in a 10-μl final volume, using 0.25 μM (each) primer (Table S1). The efficiency of primer sets was evaluated by qPCR in serial dilutions of WT cDNA. Results were normalized to 30S ribosomal protein S12 (*PA4268*) (8).

### Protein expression and purification.

The DNA fragment encoding the periplasmic copper binding loop of CopS_(34–151)_ was amplified from genomic DNA using 3′-end primers that introduced sequences encoding either a Strep tag or a His_6_ tag joined by a tobacco etch virus (TEV) cleavage site (Table S1). The His-tagged protein had a higher expression yield and was used in Cu^+^ binding experiments since this tag does not bind monovalent Cu^+^. However, the His tag binds Cu^2+^. Cleavage of the His tag was not pursued because the CopS (dimer) and the TEV have exactly the same molecular weight and it is not possible to ensure full cleavage. In consequence, a Strep-tagged protein was used in Cu^2+^ binding experiments. Resulting amplicons were cloned in the pBAD-topo vector (Invitrogen) and expressed in E. coli LMG194 cells. His-tagged CopS_(34–151)_ was purified using Ni-NTA columns (Roche) (11). Strep-tagged CopS_(34–151)_ was affinity purified using Strep-Tactin XT Superflow columns (IBA) (11). Purified proteins were stored in 20% glycerol, 25 mM Tris (pH 8), 100 mM sucrose, 150 mM NaCl at −80°C. Protein concentrations were determined in accordance with work of Bradford (76), and purity was estimated by SDS-PAGE followed by Coomassie brilliant blue staining (Fig. S7). Proteins were purified as ≥90% *apo* forms as confirmed by AAS.

### Copper binding determinations.

CopS_(34–151)_-Cu^+^ binding stoichiometry was determined by incubating CopS_(34–151)_ His-tagged protein with five times molar excess of CuSO_4_ in 25 mM HEPES, pH 8, 150 mM NaCl, 0.5 mM DTT for 10 min at room temperature with gentle agitation. DTT was included to reduce Cu^2+^ to Cu^+^ and prevent protein precipitation that occurs upon addition of excess Cu^+^ using ascorbate. This is a common observation when purified proteins are exposed to Cu and is usually solved, as in this case, by replacing the reducing agent. Unbound Cu^+^ was removed by passage through a Sephadex G-10 column (GE Healthcare) followed by two washing steps using a 3-kDa Centricon. The amount of Cu^+^ bound to protein was determined by AAS.

CopS_(34–151)_-Cu^+^ dissociation constants (*K_D_*) were determined by competition assays with the chromogenic ligands BCS {[Cu^I^(BCS)_2_]^3−^ β_2_′ formation constant 10^20.8^ M^−2^, ε_483 nm_ 13,000 M^−1^ cm^−1^} and BCA {[Cu^I^(BCA)_2_]^3−^ β_2_′ formation constant 10^17.7^ M^−2^, ε_562 nm_ 7,900 M^−1^ cm^−1^ [77]}. Cu^+^ solutions were generated from CuSO_4_ in the presence of large excess ascorbate and NaCl, which stabilizes Cu^+^ as [Cu^I^Cl_n_]^(17 −^ *^n^*^)−^ (78). Briefly, for BCS competitions, 10 μM Cu^+^, 25 μM BCS in buffer 25 mM HEPES, pH 8, 150 mM NaCl, 10 mM ascorbic acid were titrated with 10 to 50 μM His-tagged CopS_(34–151)_ and incubated for 5 min at room temperature, and the 300- to 800-nm absorption spectra were recorded. The same protocol was used for BCA competitions using 18.7 μM Cu^+^, 100 μM BCA, and 5 to 50 μM protein instead. CopS_(34–151)_-Cu^+^ *K_D_*s were calculated by curve-fitting of the experimental data to the equilibrium in equations 1 and 2 (54).

(1) $$MP+2L\prime \;⇋\;ML_{2}+P$$

(2) $$K_{D}\beta_{2}\prime =\frac{\left({\left[P\right]}_{\text{total}}/\left[\mathrm{MP}\right]\right)-1}{{\left\{\left({\left[L\right]}_{\text{total}}/\left[{\mathrm{ML}}_{2}\right]\right)-2\right\}}^{2}\left[{\mathrm{ML}}_{2}\right]}$$

CopS_(34–151)_-Cu^2+^ *K_D_*s were determined using the indicator PAR as competitor ([Cu^II^(PAR)] conditional *K_A_*′ formation constant for Cu^2+^ at pH 7.4 of 10^14.6^ M^−1^, isosbestic point *A*_445 nm_, ε_505 nm_ 41,500 M^−1^ cm^−1^ [79]). Four micromolar Cu^+^, 10 μM PAR in buffer 20 mM HEPES, pH 7.4, 150 mM NaCl were titrated with 2 to 20 μM Strep-tagged CopS_(34–151)_ and incubated at room temperature to equilibrate until no further spectral changes were observed (60 min), and the 300- to 800-nm absorption spectra were recorded. The *K_D_* value was obtained from a curve-fitting of a series of experimental data to equations 3 and 4. Reported errors are asymptotic standard errors provided by the fitting software (Kaleidagraph; Synergy).

(3) MP+L′ ⇋  ML+P

(4) $$K_{D}K_{A}\prime =\frac{\left({\left[P\right]}_{\text{total}}/\left[\mathrm{MP}\right]\right)-1}{\left({\left[L\right]}_{\text{total}}/\left[\mathrm{ML}\right]\right)-1}$$

### Bioinformatic approaches.

In general, protein sequences were retrieved from UniProt (80) and aligned using Clustal Omega (81). To build the phylogenetic trees, the full-length protein sequences of E. coli CusS and P. aeruginosa CopS sequences were independently used as query to search for homologs in the UniProtKB database using the UniProt/BLAST tool. Sequences more than 45% identical over their entire lengths were retrieved and aligned. Phylogenetic trees were calculated with the Jalview software (82), using the distance matrix BLOSUM62 and the Average Distance (unweighted pair group method using average linkages [UPGMA]) algorithm.

The structure of the soluble periplasmic copper binding loop of CopS_(34–151)_ was modeled using the online server SWISS-MODEL (83) and the structure of the E. coli CusS soluble periplasmic domain (PDB ID: 5KU5) (47) as the template. Conserved metal binding residues of CopS were identified by superimposing its structure with 5KU5 using UCSF Chimera (84).
