# Supplementary material for: The Two-Component System CopRS Maintains Subfemtomolar Levels of Free Copper in the Periplasm of Pseudomonas aeruginosa Using a Phosphatase-Based Mechanism
Source: mSphere. 2020 Dec 23;5(6):e01193-20. doi: 10.1128/mSphere.01193-20 (PMC7763554; doi:10.1128/mSphere.01193-20)
Supplement: FIG S1 [file mSphere.01193-20-sf001.pdf]

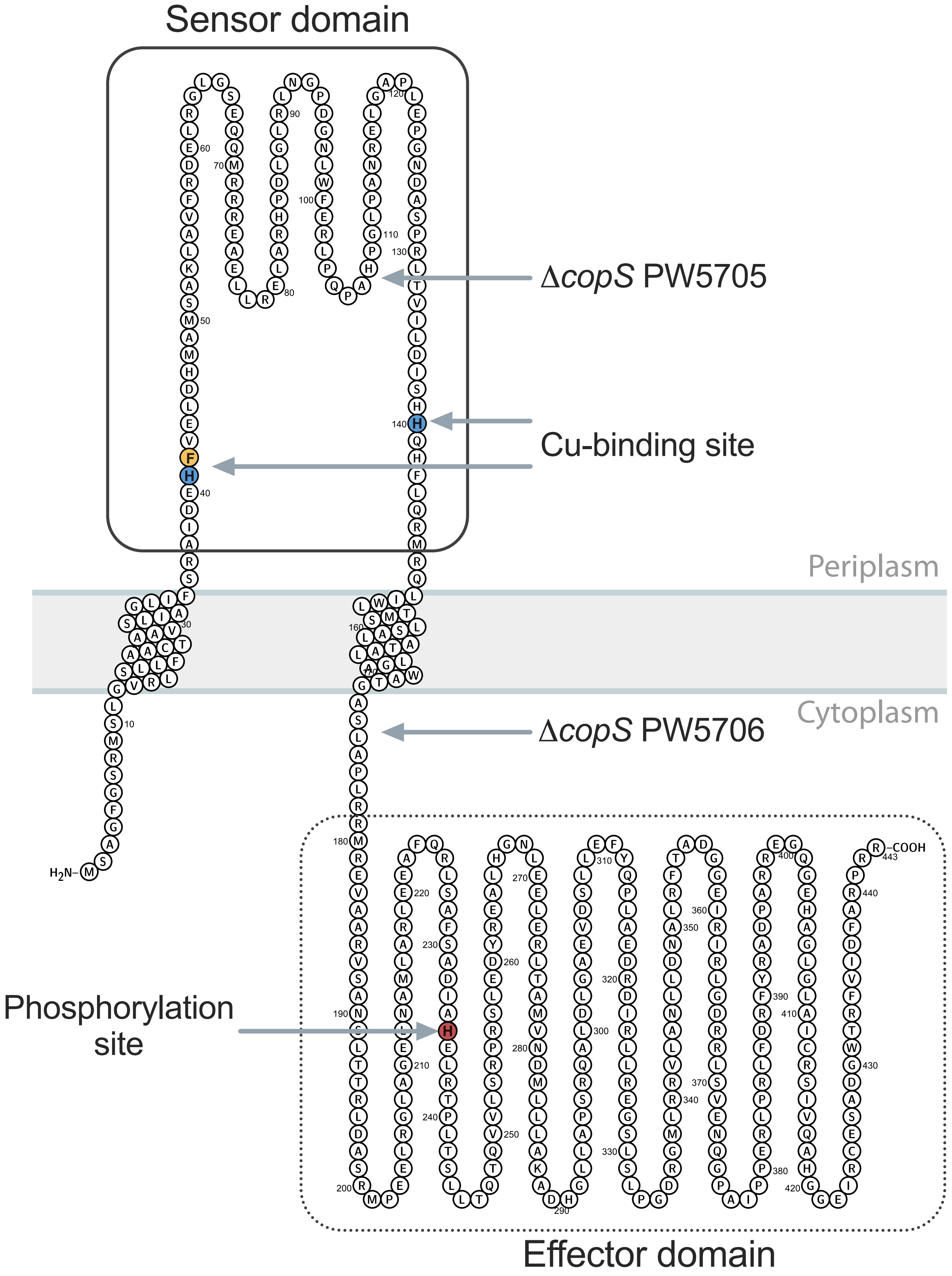

Sensor domain
ΔcopS PW5705
Cu-binding site
Periplasm
Cytoplasm
ΔcopS PW5706
Phosphorylation site
Effector domain
H2N–
–COOH
