# Supplementary figures and images for: The Two-Component System CopRS Maintains Subfemtomolar Levels of Free Copper in the Periplasm of Pseudomonas aeruginosa Using a Phosphatase-Based Mechanism
Source: mSphere. 2020 Dec 23;5(6):e01193-20. doi: 10.1128/mSphere.01193-20 (PMC7763554; doi:10.1128/mSphere.01193-20)

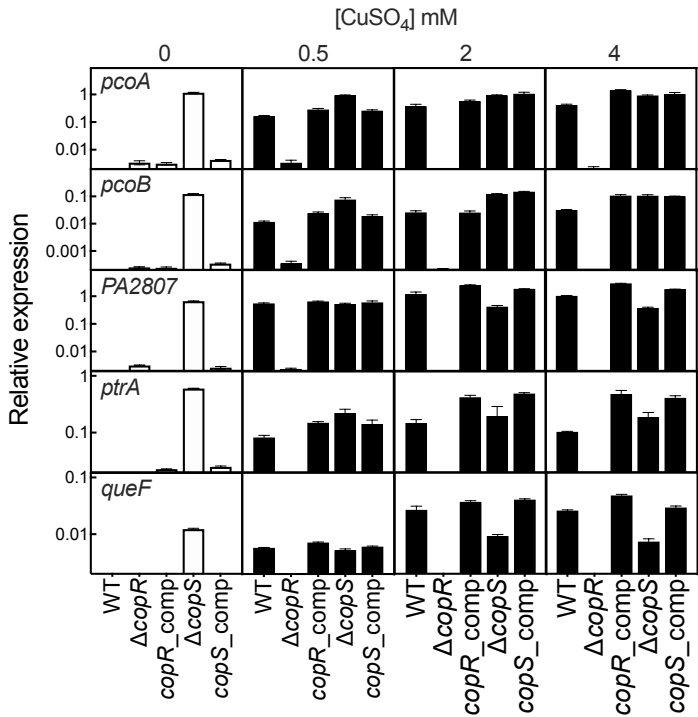

Supplement: FIG S2 [file mSphere.01193-20-sf002.pdf]

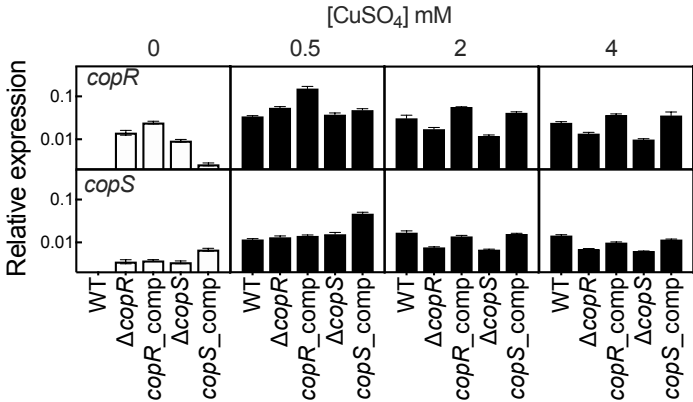

Supplement: FIG S3 [file mSphere.01193-20-sf003.pdf]

A

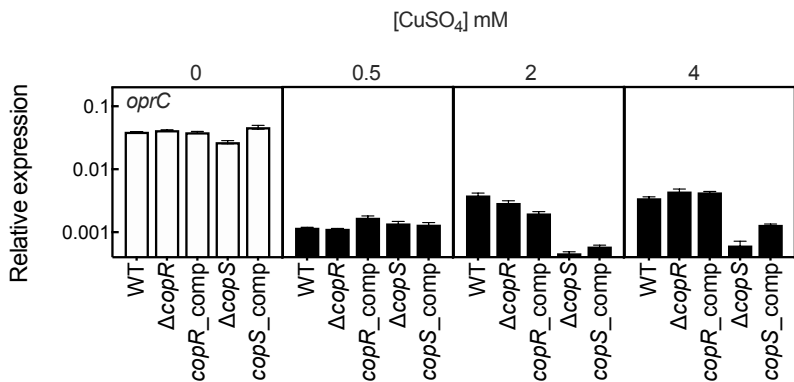

B

[CuSO<sub>4</sub>] mM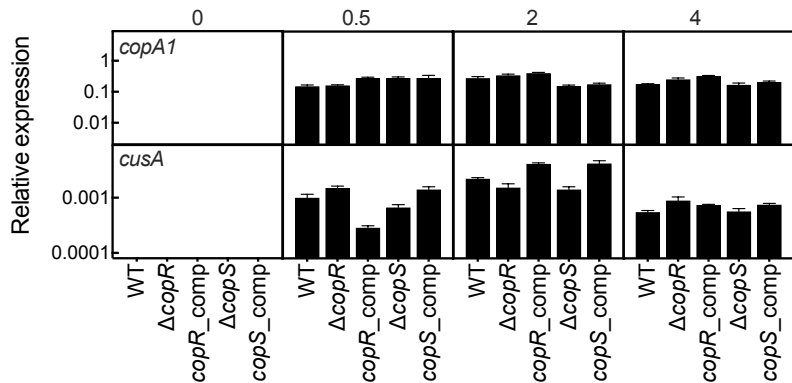

Supplement: FIG S4 [file mSphere.01193-20-sf004.pdf]

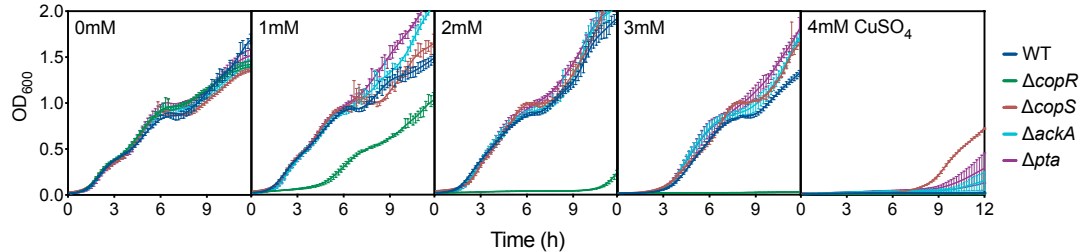

Supplement: FIG S5 [file mSphere.01193-20-sf005.pdf]

**A**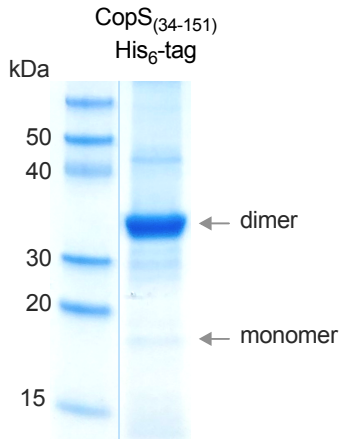**B**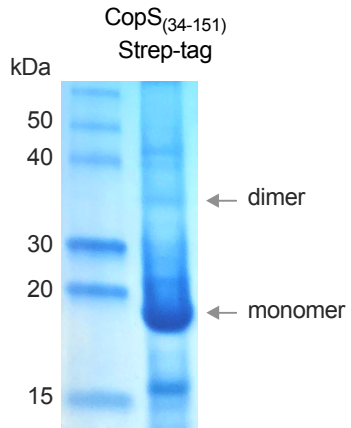

Supplement: FIG S7 [file mSphere.01193-20-sf007.pdf]

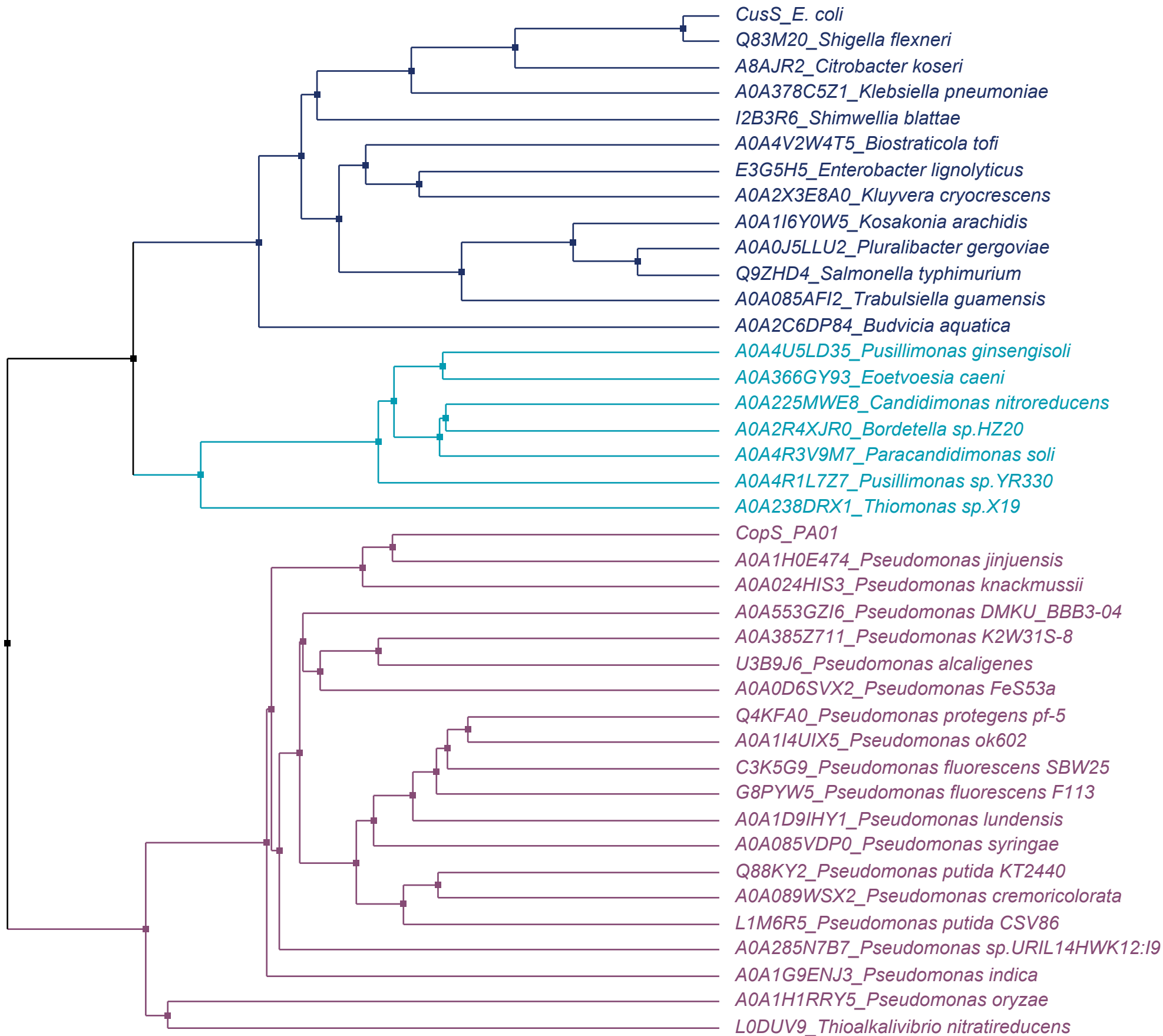

Supplement: FIG S8 [file mSphere.01193-20-sf008.pdf]
