# Supplementary material for: The Two-Component System CopRS Maintains Subfemtomolar Levels of Free Copper in the Periplasm of Pseudomonas aeruginosa Using a Phosphatase-Based Mechanism
Source: mSphere. 2020 Dec 23;5(6):e01193-20. doi: 10.1128/mSphere.01193-20 (PMC7763554; doi:10.1128/mSphere.01193-20)
Supplement: FIG S6 [file mSphere.01193-20-sf006.pdf]

A

```
CopR_PA01                               ----MKLLIVEDEPRIGQYLRQGLAEAGFAVDLSDDGNEGEQLALGGDYDLLILDVMLPG   56
P0AA16_OmpR_Escherichia_coli            MQENYKILVVDDDMRLRALLERYLTEQGFQVRSVANAEQMDRLLTRESFHLMVLDLMLPG   60
P0AE88_CpxR_Escherichia_coli            ---MNKILLVDDDRELTSLLKELLEMEGFNVIVAHDGEQALDL-LDDSIDLLLLDVMMPK   56
P0AFJ5_PhoB_Escherichia_coli            --MARRILVVEDEAPIREMVCFVLEQNGFQPVEAEDYDSAVNQLNEPWPDLILLDWMLPG   58
P0DM78_PhoP_Salmonella_typhimurium      ---MMRVLVVEDNALLRHHLKVQLQDSGHQVDAAEDAREADYYLNEHLPDIAIVDLGLPD   57
                                             ::*:*:*:  :   :   *   *.      :  .          .: ::*  :*

CopR_PA01                               RDGWQILRSVRDAGM--TVPVLFLTARDAVEDRVRGLEQGADDYLVKPFAFVELLARVRT   114
P0AA16_OmpR_Escherichia_coli            EDGLSICRRLRSQSN--PMPIIMVTAKGEEVDRIVGLEIGADDYIPKPFNPRELLARIRA   118
P0AE88_CpxR_Escherichia_coli            KNGIDTLKALR-QTH--QTPVIMLTARGSELDRVLGLELGADDYLPKPFNDRELVARIRA   113
P0AFJ5_PhoB_Escherichia_coli            GSGIQFIKHLKRESMTRDIPVVMLTARGEEEDRVRGLETGADDYITKPFSPKELVARIKA   118
P0DM78_PhoP_Salmonella_typhimurium      EDGLSLIRRWRSSDV--SLPVLVLTAREGWQDKVEVLSSGADDYVTKPFHIEEVMARMQA   115
                                         .* .  :  :        *::.:**:    *::  *. *****: ***   *::**:::

CopR_PA01                               LLRRGSQQL-------QETTLQLADLELDLLRRRVQRQGKRIDLTAKEFALLELLLRRSG   167
P0AA16_OmpR_Escherichia_coli            VLRRQANELPGA-PSQEEAVIAFGKFKLNLGTREMFREDEPMPLTSGEFAVLKALVSHPR   177
P0AE88_CpxR_Escherichia_coli            ILRRSHWSEQQQNNDNGSPTLEVDALVLNPGRQEASFDGQTLELTGTEFTLLYLLAQHLG   173
P0AFJ5_PhoB_Escherichia_coli            VMRRISPMA-------VEEVIEMQGLSLDPTSHRVMAGEEPLEMGPTEFKLLHFFMTHPE   171
P0DM78_PhoP_Salmonella_typhimurium      LMRRNSG-L-------ASQVINIPPFQVDLSRRELSVNEEVIKLTAFEYTIMETLIRNNG   167
                                        ::**             . .: .  : ::   :.     : : :   *: ::  :  .

CopR_PA01                               EVLPKSLIASQVWDMNFDSDTNVIEVAIRRLRAKVDDD-YPQRLIHTVRGMGYVLEERDE   226
P0AA16_OmpR_Escherichia_coli            EPLSRDKLMNLARGREYSAMERSIDVQISRLRRMVEEDPAHPRYIQTVWGLGYVFVPDGS   237
P0AE88_CpxR_Escherichia_coli            QVVSREHLSQEVLGKRLTPFDRAIDMHISNLRRKLPDRKDGHPWFKTLRGRGYLMVSAS-   232
P0AFJ5_PhoB_Escherichia_coli            RVYSREQLLNHVWGTNVYVEDRTVDVHIRRLRKALEPG-GHDRMVQTVRGTGYRFSTRF-   229
P0DM78_PhoP_Salmonella_typhimurium      KVVSKDSLMLQLYPDAELRESHTIDVLMGRLRKKIQAQ-YPHDVITTVRGQGYLFELR--   224
```

B

```
CopS_PA01                              ----------------MSAGFGSRMSLGVRLSLLFAACTAAVSLIAGLIFSRAIDEHFVEL   45
Q88KY2_Pseudomonas_putida              --------------------MMRRVSLGSRLALLFAACTATVSLGAGLLFSRASEQHFVEL   41
C3K5G9_Pseudomonas_fluorescens         --------------------MIKRLSLASRLALLFAACTAVVSLIAGVLFNRASEAHFIEL   41
U3B9J6_Pseudomonas_alcaligenes         ------------------------MSLANRLALLFAACTAAVALLAGALFSRASEMHFIEL   37
A0A085VDP0_Pseudomonas_syringae        --------------------MIRRWSLASRLALLFAACTAVVSLIAGVLFNQASETHFIEL   41
CusS_E.coli                            ---------------MVSKPFQRPFSLATRLTFFISLATIAAFFAFAWIMIHSVKVHFAEQ   46
Q83M20_Shigella_flexneri               ---------------MVSKPFQRPFSLATRLTFFISLATIAAFFAFAWIIIHSVKVHFAEQ   46
A8AJR2_Citrobacter_koseri              ---------------MVTKRFQRPFSLATRLTFFISLATIASFFAFAWIMIHSVKVHFAEQ   46
A0A378C5Z1_Klebsiella_pneumoniae       -------------------MAAKRPFSLATRLTFFISLATIIAFFAFTWIMIHSVKAHFEER   43
A0A4V2W4T5_Biostraticola_tofi          MPVSRSHRSFPRGIRSFLLATRPLSLAMRLTCFISLATIAAFVAFAWIMLHSVEHHFAEQ   60
                                                               **. **: ::: .*    .    :: :: . ** *

CopS_PA01                              DHMAMSAKLAVFRDELRGLGSEQQMRRREAELLRELARHPDLGLRLNGPDGNLWFERLPQ   105
Q88KY2_Pseudomonas_putida              DQQLLDSRLSLFRTQLAGVSTADELQARLPALRDELSHQADLALRISASNGATWFESRSG   101
C3K5G9_Pseudomonas_fluorescens         DQQQLDSQLVALRSTLQGVDSPELFAQREAQLRAELNRQPELALRITAQGQ-RWFDGAPG   100
U3B9J6_Pseudomonas_alcaligenes         DQQQLQGKLAVFTELLQGVTSTTALVARRPALEQELQRHPELALRIEGPDGQVWFSSRAL   97
A0A085VDP0_Pseudomonas_syringae        DQQLLESKLPGLRSLLQNVRSEQDLAGRAAQLKAELAHQPDLGLRISGPGSEPWFDSAAL   101
CusS_E.coli                            DINDLKEISATLERVLNHPDETQ--ARRLMTLEDIVSGYSNVLISLADSQGKTVYHSPGA   104
Q83M20_Shigella_flexneri               DINDLKEISATLERVLNHPDETQ--ARRLMTLEDIVSGYSNVLISLADSHGKTVYHSPGA   104
A8AJR2_Citrobacter_koseri              DINDLKEISATLERILTHPDEPE--ARRLEILKNVAAGYSNVIISLEDANQRAIFHSPGS   104
A0A378C5Z1_Klebsiella_pneumoniae       DVHDLRQLSTTLETVLDHADYPP--ARRLEIVRNIIAGYANVFICLDDGQGNILFQSPNG   101
A0A4V2W4T5_Biostraticola_tofi          DVSDLKQISATLANTLKKANEPQ--AEKVEKLRTALAGYRNIAVLLKTEDNRLLYRSADG   118
                                       *   :      :   *           :   :        :: : :        :

CopS_PA01                              PAHP----------------------------GL-------PANRELGAPLEPGNDA-SP   129
Q88KY2_Pseudomonas_putida              LPHA------------------------AQATGLATLHAPGIDYRSLSVPLTQGAMQ-SP   136
C3K5G9_Pseudomonas_fluorescens         V--N-----------------------LPTGPGLHSLQNAGTDYRVYNTPLRANQPD-SP   134
U3B9J6_Pseudomonas_alcaligenes         PDSL-----------------------PHTQ----HWNHQDTAYRVMSTK-----QG-DL   124
A0A085VDP0_Pseudomonas_syringae        KPSL-----------------------PVTDEGLHTLISDQTAYRTYTVKLDPATPD-SP   137
CusS_E.coli                            PDIREFTRDAIPDKDAQGGEVYLLSGPTMMMPGHGHGHMEHSNWRMINLPVGPLVDG-KP   163
Q83M20_Shigella_flexneri               PDIREFARDAIPDKDARGGEVYLLSGPTIMMPGHGHGHMEHSNWRMINLPVGPLVDG-KP   163
A8AJR2_Citrobacter_koseri              PDLRQFIAKATPDSSARNGDVFLLSSPDLQSKRHANGHGTHSAWRMIRLPVGQLADG-QP   163
A0A378C5Z1_Klebsiella_pneumoniae       PDLSHILSTPGLAMQLRDGNVISWTDPQPRAMAHDNHPMETRAWRLIMLPLGKQADG-KP   160
A0A4V2W4T5_Biostraticola_tofi          PDLDAIGSSPAFAANLASREVFTWSDTQSTQSAHGDS-AKHPAYRVIASTVQTRVAGKVT   177
                                                                           *

CopS_PA01                              RL--TVILDISHHQHFLQRMRQLIWLTMSLSALATALLGAWATGASLAPLRRMREVAARV   187
Q88KY2_Pseudomonas_putida              RL--TLYLDITHHQHFLQGMQRLIWLTVGLSALITALLGAWAARSGLRPLRQMGQVAASV   194
C3K5G9_Pseudomonas_fluorescens         QL--TLVLDITHHQHFLQRMQHLIWLTVGLSALATALLGAWAARSGLRPLRRMGEVAASV   192
U3B9J6_Pseudomonas_alcaligenes         RL--TLLLDITHHQHFLEQMQRLIWLCVGLSALATALLGAWVARRGLRPLRQMTRVTQQV   182
A0A085VDP0_Pseudomonas_syringae        QL--TLLLDITHHQHFLQRMQRLIWLTVGLSALATALLGAWAARSGLRPLHNMSRIAASV   195
CusS_E.coli                            IYTLYIALSIDFHLHYINDLMNKLIMTASVISILIVFIVLLAVHKGHAPIRSVSRQIQNI   223
Q83M20_Shigella_flexneri               IYTLYIALSIDFHLHYINDLMNKLIMTASVISILIVFIVLLAVHKGHAPIRSVSRQIQNI   223
A8AJR2_Citrobacter_koseri              AYTLYMALSIDFHLHYINDLKNKLIMTASLISMMIIFIVLFAVYKGHEPIRSVSRRIQNI   223
A0A378C5Z1_Klebsiella_pneumoniae       AYHLLMALSIDFHLHYINELKAKLISAASIISLLIIAIVLFVVYQGHKPIRQISRQIQNI   220
A0A4V2W4T5_Biostraticola_tofi          RYSLMIALSIDFHLHYIEELKHNLIMIAAGISLLIVFIVLFAVYKGHTPLRNVSKKIKNI   237
                                            : *.* .* *:::  :   :    .  ::    :   ..  .  *:: : .    :

CopS_PA01                              SANSLTTRLDASRMPEELRGLAGELNAMLARLEEAFQRLSAFSADIAHELRTPLTSLLTQ   247
Q88KY2_Pseudomonas_putida              SARSLTTRLPVAQMPEELAELASSMNAMLQRLDDAFQRLSAFSADIAHELRTPLSNLLTH   254
C3K5G9_Pseudomonas_fluorescens         SAHSLTQRLPQQHMPVELAELAQTFNAMLSRLDDAFQRLSAFSADIAHELRTPLSNLLTQ   252
U3B9J6_Pseudomonas_alcaligenes         SASSLTARLPADALPAELGELAASFNAMLARLEDAFARLSAFSADIAHELRTPLSNLLTQ   242
A0A085VDP0_Pseudomonas_syringae        SASSLTTRLPQEQMPAELAELTQAFNGMLGRLDDGFQRLSAFSADIAHELRTPLSNLLTH   255
CusS_E.coli                            TSKDLDVRLDPQTVPIELEQLVLSFNHMIERIEDVFTRQSNFSADIAHEIRTPITNLITQ   283
Q83M20_Shigella_flexneri               TSKDLDVRLDPQTVPIELEQLVLSFNHMIERIEDVFTRQSNFSADIAHEIRTPITNLITQ   283
A8AJR2_Citrobacter_koseri              TSKDLDVRLDPQAVPIELERLVLSFNHMLERIEDVFTRQSNFSADIAHEIRTPITNLVTQ   283
A0A378C5Z1_Klebsiella_pneumoniae       TSRDLDVRLDPQAVPVELERLALSFNHMLERIEDVFTRQSNFSADIAHEIRTPITNLVTQ   280
A0A4V2W4T5_Biostraticola_tofi          TSENLDVRLDPHKVPIELEQLVISFNNMIERIEDVFTRQANFSADIAHEIRTPITNLITQ   297
                                       :: .*  **    :* **   *.  :* *: *::: * * : ********:***::.*:*:

CopS_PA01                              TQVVLSRPRSLEDYREALHGNLEELERLTAMVNDMLLLAKADHGLLAPSRQALDLGAEVD   307
Q88KY2_Pseudomonas_putida              TQVTLTRPRSLEEYREALHGNLEELQWMAQMINDMLFLAKADHGLLVPGDAPLALHDEVD   314
C3K5G9_Pseudomonas_fluorescens         TQVILTQPRPLEDYREALHSNLEELQWMAQLVNDMLYLAKADHGLLVPKREPLALADEVE   312
U3B9J6_Pseudomonas_alcaligenes         TQVILSQPRALEDYQEALHSNLEELQHLAQMVGDMLLLAKADNGLLQTRRETLALERELT   302
A0A085VDP0_Pseudomonas_syringae        TQVTLTRPRDIEDYREALLSNLEELQSMAQMVNDMLYLAKAEHGLLTPTCERLRLEDEVQ   315
CusS_E.coli                            TEIALSQSRSQKELEDVLYSNLEELTRMAKMVSDMLFLAQADNNQLIPEKKMLNLADEVG   343
Q83M20_Shigella_flexneri               TEIALSQSRSQKELEDVLYSNLEELTRMAKMVSDMLFLAQADNNQLIPEKKMLNLADEVG   343
A8AJR2_Citrobacter_koseri              TEIALSQTRSQKELEDVLYSNLEEFGRMSKMVSDMLFLAQADNNQLIPEKKALDLADEVH   343
A0A378C5Z1_Klebsiella_pneumoniae       TEIALSQSRSQQELEEVLYSNLEEFSRMSRMVSDMLFLAQADNNQLIPEQQALDLADEVH   340
A0A4V2W4T5_Biostraticola_tofi          TEIVLRQPRTIKELEDVLYSNLEEFSHMAKMVSDMLFLAQADNQQLIPERSLIDLETEVR   357
                                       *:: * : *  :: .:.* .****:  :: ::.*** **:*::  *       : *  *:
```

```
CopS_PA01                             SLLEFYQPLAEDRDIRLLREGSL-SLPGDRGMLRRVLANLLDNALRFTADGGEIRIRLGD    366
Q88KY2_Pseudomonas_putida             ALLEYYAPLAEDSDVQMLREGEA-VLHGDQHMLRRALSNLLDNAMRFTPAGGQIKVTLGP    373
C3K5G9_Pseudomonas_fluorescens        ALLEFFALLAEDAHVSLVREGTA-HTMGDRGMLRRALSNLLDNALRFTPAGGEVRVRMVD    371
U3B9J6_Pseudomonas_alcaligenes        ALAEYFTPLAEEAGVHLHVDGQA-ALSADRALLHRALSNLIDNALRFTPRGGELHLSSAQ    361
A0A085VDP0_Pseudomonas_syringae       TVLEFFAPLAEDAQVTLSSDGHA-SIDGDRTMLRRVLSNLLDNAVRHTPAGGYVKVTLSE    374
CusS_E.coli                           KVFDFFEALAEDRGVELRFVGDKCQVAGDPLMLRRALSNLLSNALRYTPTGETIVVRCQT    403
Q83M20_Shigella_flexneri              KVFDFFEALAEDRGVELRFVGDKRQVAGDPLMLRRALSNLLSNALRYTPTRETIVVRCQT    403
A8AJR2_Citrobacter_koseri             KVFDFFEAWAEEREVRLHFEGRACWVSGDPIMLRRALSNLLSNAMRYTPKGEAVTVRLKE    403
A0A378C5Z1_Klebsiella_pneumoniae      KVFEFFEAWAEEKAVALRFVGSHCRVIGDPLMLRRAISNLLSNAIRYTPAGQAVTIQLSE    400
A0A4V2W4T5_Biostraticola_tofi         KVFEFFEAWADEREVRLNITGRAQPIEGDPLMLRRAISNLLSNAVRYTPPGNTIIVQLTE    417
                                       : :::   *::  : :   *      .*  :*:*.::**:.**:*.*     : :

CopS_PA01                             ----RRLSVENQGPAIPPERLPRLFDRFYRADPARREGQGEHAGLGLAICRSIVQAHGGE    422
Q88KY2_Pseudomonas_putida             ---GPTINVANTGLAIDPAALPRLFDRFYRVDPARREGSSEHAGLGLAITRSIVQAHGGC    430
C3K5G9_Pseudomonas_fluorescens        ---GVTLTVENTGAGIPAQLLPRLFDRFYRADPARHEGSSEHAGLGLAITQSIVRAHGGR    428
U3B9J6_Pseudomonas_alcaligenes        QGAKVRIEVANQGPEIPLDLRERLFDRFYRADPARREGGAEHAGLGLAIARSIVQAHGGA    421
A0A085VDP0_Pseudomonas_syringae       VDKRARINVENSGADIPESLLPRLFDRFYRADQARSE-SREHAGLGLAITRSIIQAHGGT    433
CusS_E.coli                           VDHLVQVIVENPGTPIAPEHLPRLFDRFYRVDPSRQR-KGEGSGIGLAIVKSIVVAHKGT    462
Q83M20_Shigella_flexneri              VDHQVQVSVENPGTPIAPEHLPRLFDRFYRVDPSRQR-KGEGSGIGLAIVKSIVVAHKGT    462
A8AJR2_Citrobacter_koseri             MDRQAQITVENPGAPIAPEHLPRLFDRFYRVDPSRQR-KGEGSGIGLAIVKSIVIAHQGT    462
A0A378C5Z1_Klebsiella_pneumoniae      SAETVRLVVENPGTPIAAEHLPRLFDRFYRVDPSRQR-KGEGSGIGLAIVKSIVGAHHGS    459
A0A4V2W4T5_Biostraticola_tofi         RDQWVEIQVENPGAMIAEQHLPRLFDRFYRADPSRQK-KGEGSGIGLAIVKSIVTAHQGK    476
                                            : * * *  *      ********.* :* .   * :*:**** :**: ** *

CopS_PA01                             IRCESADGWTRFVIDFARPRR  443
Q88KY2_Pseudomonas_putida             IRAECEGGWTRFVIEFTQDR-  450
C3K5G9_Pseudomonas_fluorescens        IYCESGAGWTRFVIELPAGD-  448
U3B9J6_Pseudomonas_alcaligenes        IRCESAEGWTRFILEFPA---  439
A0A085VDP0_Pseudomonas_syringae       IRCESKEGVTRFLIELPIQK-  453
CusS_E.coli                           VAVTSDARGTRFVITLPA---  480
Q83M20_Shigella_flexneri              VAVTSDARGTRFVITLPA---  480
A8AJR2_Citrobacter_koseri             VSVTSDLRATRFILTLPKHGD  483
A0A378C5Z1_Klebsiella_pneumoniae      VAAQSDLRSTRFIVVLPK---  477
A0A4V2W4T5_Biostraticola_tofi         ISVSSDAVSTKFSLSLPKRAS  497
                                      :   .    *:* : :
```
