# Supplementary material for: The Two-Component System CopRS Maintains Subfemtomolar Levels of Free Copper in the Periplasm of Pseudomonas aeruginosa Using a Phosphatase-Based Mechanism
Source: mSphere. 2020 Dec 23;5(6):e01193-20. doi: 10.1128/mSphere.01193-20 (PMC7763554; doi:10.1128/mSphere.01193-20)
Supplement: TABLE S1 [file mSphere.01193-20-st001.pdf]

Table S1. Bacterial strains, plasmids and primers used in this study

Strains	Relevant features	Reference
Pseudomonas aeruginosa		
PAO1	Wild type	(1)
Δ*copA1*	PW7626, *copA1*(PA3920)::ISphoA/hah::Tet^R	(2)
Δ*copR*	PW5704, *copR*(PA2809)::IslacZ/hah::Tet^R	(2)
copRcomp	Δ*copR* complemented with *copR* gene under endogenous promoter; C-term His_6; Gm^R	This study
$copR_{D51A}$	Δ*copR* complemented with *$copR_{D51A}$* gene under endogenous promoter; C-term His_6; Gm^R	This study
$copR_{D51A}$	Δ*copR* complemented with *$copR_{D51E}$* gene under endogenous promoter; C-term His_6; Gm^R	This study
Δ*copS*	PW5705, *copS*(PA2810)::IsphoA/hah::Tet^R	(2)
Δ*copS*	PW5706, *copS*(PA2810)::IsphoA/hah::Tet^R	(2)
copScomp	Δ*copS* complemented with *copS* gene under endogenous promoter; C-term His_6; Gm^R	This study
$copS_{H235A}$	Δ*copS* complemented with *$copS_{H235A}$* gene under endogenous promoter; C-term His_6; Gm^R	This study
Escherichia coli		
Top10	*F^- mcrA Φ80lacZΔM15 ΔlacX74 recA1 araD139 Δ(ara-leu)7697 galU galK rpsL (Str^R) endA1 nupG*	Invitrogen
LMG194	*F- ΔlacX74 gal E thi rpsL ΔphoA (Pvu II) Δara714 leu::Tn10*	Invitrogen
SM10(λpir)/pTNS2	*thi-1, thr, leu, tonA, lacY, supE, recA::RP4-2- Tc::Mu, pir / pTNS2* (Amp^R *oriR6K, tnsABCDE*); Km^R/Helper plasmid (TnsABCD site), Amp^R	(3)
HB101/pRK2013	*F- mcrB mrr hsdS20(rB- mB-) recA13 leuB6 ara-14 proA2 lacY1 galK2 xyl-5 mtl-1 rpsL20 (Sm^R) glnV44 λ- / pRK2013* (Km^R *oriColE1 RK2-Mob+ RK2-Tra+*)	(3)
**Plasmids**	**Relevant features**	**Reference**
pBADtopo	*araBAD* promoter, V5 epitope tag, His_6-tag, Amp^R	Invitrogen
$CopS_{(34-151)}$_His_6:pBAD	DNA fragment encoding for the periplasmic copper binding loop of CopS residues 34 to 151. C-term His_6-tag, Amp^R	This study
$CopS_{(34-151)}$_Strep:pBAD	DNA fragment encoding for the periplasmic copper binding loop of CopS residues 34 to 151. C-term Strep-tag, Amp^R	This study
pUC18_mini_Tn7T_Gm	Suicide delivery vector, Tn7 transposable site, Gm^R	(4)
500prom_copR: pUC18_miniTn7T_Gm	500 bp upstream of *copR* gene + *copR* gene_His_6 cloned into the suicide delivery vector, Gm^R	This study
500prom_$copR_{D51A}$: pUC18_miniTn7T_Gm	500 bp upstream of *copR* gene + *$copR_{D51A}$* gene_His_6 cloned into the suicide delivery vector, Gm^R	This study
500prom_$copR_{D51E}$: pUC18_miniTn7T_Gm	500 bp upstream of *copR* gene + *$copR_{D51E}$* gene_His_6 cloned into the suicide delivery vector, Gm^R	This study
500prom_copS: pUC18_miniTn7T_Gm	500 bp upstream of *copR* gene + *copS* gene_His_6 cloned into the suicide delivery vector, Gm^R	This study
500prom_$copS_{H235A}$: pUC18_miniTn7T_Gm	500 bp upstream of *copR* gene + *$copS_{H235A}$* gene_His_6 cloned into the suicide delivery vector, Gm^R	This study
**Primers**	**Sequence (5'-3')**	**Gene**
qPCR		
qPA4268_F	gcaaaactgcccgcaacgtc	housekeeping
qPA4268_R	tacacgaccgccacggatca	
qPA2064_F	aggtcaacctgtacggcagg	*pcoB*
qPA2064_R	aactcgcgacggatctcatagcg	

Primers	Sequence (5'-3')	Gene
qPA2065_F	caagcacaccatcgacatgcc	*pcoA*
qPA2065_R	atgcgttgtctccttcgtctaccc	
qPA2806_F	ctgtttcccatttcacgcac	*queF*
qPA2806_R	gtagcagttccagatatccacg	
qPA2807_F	cgatggagattccccgta	*PA2807*
qPA2807_R	cgaggacgatcagggaag	
qPA2808_F	ggcgacatcgtcttcgag	*ptrA*
qPA2808_R	tccttcttcgtcgcttcg	
qPA2809_F	tgctcgaactgctgctgc	*copR*
qPA2809_R	acctcgatgacgttggtatcgc	
qPA2810_F	agatacgtatccgtctcggcgacc	*copS*
qPA2810_R	acgatagaaccggtcgaacaggc	
qPA3522_F	agtacaacagctggctgttgcc	*cusA*
qPA3522_R	tggacgaacacgttgttgtcgc	
qPA3920_R	gaaacggtgctggcgaagat	*copA1*
qPA3920_R	ttaaccagggcctgctccag	
qPA3790_F	gacggctcgcagttcaag	*oprC*
qPA3790_R	gctgacgttcgatttgacg	
Mutants complementation		
pUC_copR_His_F	aactgcagtgtcgggagcattacatc	
pUC_copR_His_R	ggactagttcaatggtgatggtgatgatgggactgaaaatacaggttttcgccgctgctttcgtcgcgctcttcgag	
copSR_promot_GA_F	catgagctcattttctgcagtgtcgggagcattacatc	
copS_prom_over_R	acatccgcgagccgaacccggcgctcatgttcatccctcgttacatt	
copS_whole_F	agcgccgggttcggctcg	
copS_pUC_His_GA_R	taccgggcccttttaagctttcaatgatgatgatgatgatgtcggcgcgggcgagcgaa	
pUCTn7Gm_GA_F	aagcttaaaagggcccggtacctcgcga	
pUCTn7Gm_GA_R	ctgcagaaaatgagctcatgcatgatcg	
Site mutants		
copS_H235A_F	gcagagttgcgcaccccgctcaccagc	
copS_H235A_R	agcggggtgcgcaactctgcggcgatgtcggcggagaac	
copR_D51A_F	gcagtcatgctgcccggccgtgac	
copR_D51A_R	cggccgggcagcatgactgcgaggatcagcaggtcgtagtcg	
copR_D51E_F	gaagtcatgctgcccggccgtgac	
copR_D51E_R	cggccgggcagcatgacttcgaggatcagcaggtcgtagtcg	
Protein expression		
copSsol_Strep_F	ttcagcagggccatcgac	
copSsol_Strep_R	tcatttttcgaactgcgggtggctccaagcgctcaactggcgcatgcgttg	
copSsol_TEV_His_R	ggactgaaaatacaggttttcgccgctgctcaactggcgcatgcgttg	

1. Winsor GL, Griffiths EJ, Lo R, Dhillon BK, Shay JA, Brinkman FS. 2015. Nucleic acids research 44:D646-D653.
2. Jacobs MA, Alwood A, Thaipisuttikul I, Spencer D, Haugen E, Ernst S, Will O, Kaul R, Raymond C, Levy R, Chun-Rong L, Guenthner D, Bovee D, Olson MV, Manoil C. 2003. Proc Nat Acad Sci USA 100:14339-44.
3. Choi KH, Schweizer HP. 2006. Nat Protoc 1:153-61.
4. Choi K-H, Gaynor JB, White KG, Lopez C, Bosio CM, Karkhoff-Schweizer RR, Schweizer HP. 2005. Nature Methods 2:443.
